# LncRNA LINC00942 promotes chemoresistance in gastric cancer by suppressing MSI2 degradation to enhance *c‐Myc* mRNA stability

**DOI:** 10.1002/ctm2.703

**Published:** 2022-01-24

**Authors:** Yiran Zhu, Bingluo Zhou, Xinyang Hu, Shilong Ying, Qiyin Zhou, Wenxia Xu, Lifeng Feng, Tianlun Hou, Xian Wang, Liyuan Zhu, Hongchuan Jin

**Affiliations:** ^1^ Laboratory of Cancer Biology, Key Laboratory of Biotherapy in Zhejiang Province Cancer Center of Zhejiang University, Sir Run Run Shaw Hospital, School of Medicine, Zhejiang University Hangzhou China; ^2^ Department of Medical Oncology, Sir Run Run Shaw Hospital, School of Medicine Zhejiang University Hangzhou China; ^3^ Department of Clinical Medicine Wenzhou Medical University Wenzhou China

**Keywords:** chemoresistance, *c*‐*Myc*, LINC00942 (LNC942), m^6^A, Musashi2 (MSI2)

## Abstract

**Background:**

Chemoresistance to cisplatin (DDP) remains a major challenge in advanced gastric cancer (GC) treatment. Although accumulating evidence suggests an association between dysregulation of long non‐coding RNAs (lncRNAs) and chemoresistance, the regulatory functions and complexities of lncRNAs in modulating DDP‐based chemotherapy in GC remain under‐investigated. This study was designed to explore the critical chemoresistance‐related lncRNAs in GC and identify novel therapeutic targets for patients with chemoresistant GC.

**Methods:**

Chemoresistance‐related lncRNAs were identified through microarray and verified through a quantitative real‐time polymerase chain reaction (qRT‐PCR). Proteins bound by lncRNAs were identified through a human proteome array and validated through RNA immunoprecipitation (RIP) and RNA pull‐down assays. Co‐immunoprecipitation and ubiquitination assays were performed to explore the molecular mechanisms of the Musashi2 (MSI2) post‐modification. The effects of LINC00942 (LNC942) and MSI2 on DDP‐based chemotherapy were investigated through MTS, apoptosis assays and xenograft tumour formation in vivo.

**Results:**

LNC942 was found to be up‐regulated in chemoresistant GC cells, and its high expression was positively correlated with the poor prognosis of patients with GC. Functional studies indicated that LNC942 confers chemoresistance to GC cells by impairing apoptosis and inducing stemness. Mechanically, LNC942 up‐regulated the MSI2 expression by preventing its interaction with SCF^β‐TRCP^ E3 ubiquitin ligase, eventually inhibiting ubiquitination. Then, LNC942 stabilized *c‐Myc* mRNA in an N6‐methyladenosine (m^6^A)‐dependent manner. As a potential m^6^A recognition protein, MSI2 stabilized *c‐Myc* mRNA with m^6^A modifications. Moreover, inhibition of the LNC942‐MSI2‐c‐Myc axis was found to restore chemosensitivity both in vitro and in vivo.

**Conclusions:**

These results uncover a chemoresistant accelerating function of LNC942 in GC, and disrupting the LNC942‐MSI2‐c‐Myc axis could be a novel therapeutic strategy for GC patients undergoing chemoresistance.

## Graphical abstract

1. LNC942 is a vital chemoresistant‐related lncRNA in gastric cancer. 2. LNC942 prevents β‐Trcp‐mediated MSI2 degradation. Then, MSI2 recognizes and stabilizes the *c‐Myc* mRNA in an m^6^A‐dependent manner. 3. Treatment with MSI2 inhibitor FK228 could overcome LNC942‐induced cisplatin resistance in gastric cancer.

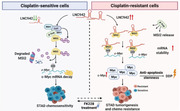

## 1 INTRODUCTION

Despite technological advances for the early diagnosis of cancers, gastric cancer (GC), an extremely aggressive tumor, remains the fifth leading cause of cancer‐related death worldwide.^1^ Chemotherapy is one of the principal treatment patterns for GC; cisplatin (DDP), either alone or combined with other chemotherapeutic agents, is the standard first‐line chemotherapy for advanced GC.^2^, ^3^ Although the combination of DDP with fluoropyrimidine demonstrated short median survival, DDP alone cannot markedly enhance the overall prognosis of patients with GC, which can be ascribed to primary and/or acquired resistance and the associated dose‐limiting side‐effects.^4^ Therefore, identifying genetic determinants of chemosensitivity and developing new agents to synergize with chemotherapeutics, including DDP, is imperative for GC treatment.

Long non‐coding RNAs (lncRNAs) represent a heterogeneous class of transcripts >200 nucleotides (nt), having no or deficient protein‐coding ability.^5^ Accumulating evidence suggests an association of lncRNA dysregulation with tumourigenesis, progression and chemoresistance.^6^, ^7^ Mechanically, lncRNAs can regulate chemotherapy resistance by interacting with DNAs, RNAs and proteins to influence their expressions and/or functions.^7^ In addition, lncRNAs can modulate autophagy, apoptosis and cancer cell stemness‐associated signaling pathways.^8^ Therefore, targeting lncRNAs may be a promising approach to restore chemosensitivity and enhance chemotherapy efficacy in GC.

Musashi2 (MSI2) is a member of RNA‐binding proteins (RBPs), which plays prominent roles as an oncoprotein in various types of tumours, including leukemia, glioblastomas, pancreatic, breast, lung and colorectal cancers. It participates in vital oncogenic signaling pathways (e.g., NOTCH, Ras/MAPK, TGF‐β/SMAD3), cancer initiation and progression through binding and regulating the mRNA stability and translation.^9^ Moreover, MSI2 has been reported to maintain liver cancer stem cells chemoresistance and stemness through activating LIN28A.^10^ However, inadequate information is available about the regulation of MSI2 protein and the functional contribution of MSI2 to drug resistance in GC.

In this study, differentially expressed lncRNAs microarray revealed that LNC942 is the most significantly up‐regulated lncRNA in chemoresistant GC cells. LNC942 could facilitate chemoresistance in GC by suppressing DDP‐induced apoptosis and inducing stemness features. More specifically, LNC942 interacts with MSI2, prevents SCF^β‐TRCP^ E3 ubiquitin (Ub) ligase‐mediated MSI2 ubiquitination and inhibits subsequent proteasomal degradation. Moreover, MSI2 was found to function as an m^6^A recognition protein to promote the stability and expression of its target mRNAs, such as *c‐Myc*. The inhibition of the LNC942‐MSI2‐c‐Myc axis was found to effectively abrogate chemoresistance of GC cells in vitro and in vivo. Collectively, our study extends the current understanding of the lncRNA‐mediated protein ubiquitination in the chemoresistance of GC, highlighting the potential role of MSI2 as a new m^6^A reader controlling mRNA decay and contributing to the identification of effective therapeutic targets in overcoming chemoresistance.

## 2 MATERIALS AND METHODS

### 2.1 Cell culture, reagents and antibodies

Human GC cell lines, SGC7901, BGC823, and human 293T cell line were purchased from the Cell Bank of the Chinese Academy of Sciences (Shanghai, China). Chemoresistant SGC7901 and BGC823 cells (SGC‐R and BGC‐R) were established in our laboratory.^11^ All the aforementioned GC cells were cultured in RPMI1640 medium (Invitrogen, USA), and 293T cells were cultured in dulbecco's modified eagle medium (DMEM) (Invitrogen, USA). All the mediums used in the experiment were added with an extra 10% fetal bovine serum (FBS).

The chemical reagents used in this study included MG‐132 (Sigma‐Aldrich, 474790), MLN4924 (ApexBio, B1036), Actinomycin D (ActD) (Sigma‐Aldrich, 129935) and cycloheximide (CHX) (Sigma‐Aldrich, C7698). DDP (MCE, HY‐17394), Romidepsin (FK228) (MCE, HY‐15149) and 10058‐F4 (Selleck, S7153) were used as the chemotherapeutics.

Commercially available antibodies used are as follows: anti‐α‐tubulin (Sigma‐Aldrich, t5168), anti‐MSI2 (Novus, NBP2‐19443), anti‐c‐Myc (CST,5605s), anti‐Flag‐tag (Sigma‐Aldrich, F1804‐1), anti‐HA‐tag (Roche, 11666606001), anti‐Ub (Santa cruz, sc‐8071), anti‐Cullin1 (Santa cruz, sc11384), anti‐β‐Trcp (CST, 4394S), anti‐FBXW7 (ABNOVA, H00055294‐M03), anti‐biotin, Horseradish peroxidase (HRP)‐linked (CST, 7075), anti‐YTHDF2 (Proteintech, 24744‐1‐AP), anti‐METTL3 (Abclonal, a8370), anti‐METTL14 (Abclonal, a8530), anti‐WTAP (Abcam, ab195380), anti‐IGF2BP1 (CST, 8482S), anti‐IGF2BP2 (CST, 14672S), anti‐IGF2BP3 (BETHYL, A303‐426A), anti‐cleaved‐PARP1 (CST, 9541S), anti‐cleaved‐caspase 3 (CST, 9661S) and anti‐m^6^A (Abcam, ab208577) antibodies.

### 2.2 Lentiviral infection and transient transfection

All of the small interfering RNAs (siRNAs) were produced by GenePharma (Shanghai, China) and transiently transfected in cells with Lipofectamine RNAiMAX Transfection Reagent (ThermoFisher, USA). The Flag‐MSI2 and related MSI2 truncation, Myc‐MSI2 and Flag‐c‐Myc plasmids were generated by PCR and inserted into indicated vectors. Flag/HA‐β‐Trcp,^12^ His‐Ub, Flag/HA‐FBXW7^13^ and Flag‐Cullin family members (1, 3, 4B, 5)^14^ were constructed as described previously. The indicated plasmids were used to be transfected with Lipofectamine 2000 (Invitrogen, USA). The siRNAs and plasmids sequences are summarized in Tables S1 and S2.

The lentiviral of lncRNA LNC942 overexpressing construct was yielded by PCR and ligated into a pCDH vector. Then, cells were infected with lentiviral using polybrene (8 μg/ml) for 12 h and selected by puromycin for 14 days.

### 2.3 RNA extraction and quantitative real‐time PCR

Trizol reagent (Invitrogen, USA) was applied to extract total RNA, and the NanoDrop 2000 (ThermoFisher, USA) was applied for RNA concentration quantification. High‐capacity cDNA Reverse Transcription Kit (Applied Biosystems, USA) was applied for the reverse transcription of cDNA. The quantitative real‐time PCR (qRT‐PCR) was performed using SYBR Green Kit (Thermo Fisher, USA) and Light Cycler 480 Ⅱ system (Roche, China). β‐actin or 18S rRNAs were adopted for the relative target RNA expression normalization with the 2 ^−ΔΔct^ method.^15^ The primers are listed in Table S3.

### 2.4 Subcellular fractionation

Nuclear and cytoplasmic RNA was isolated with the nuclear or cytoplasmic RNA Purification Kit (ThermoFisher Scientific, USA). Cells were firstly lysed on ice for 10 min with cell fraction buffer. The cytoplasmic and nuclear fractions were separated into the supernatant and precipitation respectively, by a 5‐min centrifugation at 500 g. The above‐obtained RNA was extracted and subsequently analysed by qRT‐PCR. Gyceraldehyde‐3‐phosphate dehydrogenase (GAPDH) and Malat1 were adopted for cytoplasmic and nuclear markers, respectively.

### 2.5 RNA fluorescence in situ hybridization and immunofluorescence

For RNA fluorescence in situ hybridization (FISH) assays, the pre‐seeded cells first were fixed with 4% paraformaldehyde for 10 min and permeabilized with .1% Triton X‐100 for 10 min. Then, the cells were bound to the Cy3‐labelled lncRNA LNC942 probes (GenePharma, China)) overnight at 37 °C. DAPI (Sigma‐Aldrich, USA) was used to stain the nuclei before cells were sealed onto the glass slides.

For immunofluorescence (IF) assays, cells were seeded, fixed and permeabilized under the same condition as the RNA FISH assays. After that, cells were incubated with the MSI2 antibody (Abcam, ab76148, 10 μg/ml) overnight at 4°C and incubated with the Alexa Fluor 488 goat anti‐rabbit IgG (H+L) secondary antibody (Abcam, ab150077, 5 μg/ml) for 1 h. All images were visualized and filmed using an Olympus FV1200 SPECTRAL Laser scanning Confocal Microscope.

### 2.6 RNA immunoprecipitation assay

RNA immunoprecipitation (RIP) assays were conducted using Magna RIP^TM^ RBP Immunoprecipitation Kit (Millipore, No.17‐700). In brief, 1 × 10^7^ cells were lysed with 100 μl RIP lysis buffer and incubated with indicated antibodies and protein A/G magnetic beads overnight at 4°C. The recovered RNA‐protein complex was washed six times with wash buffer and protein denaturation at 55°C. Extracted RNA was submitted to qRT‐PCR analysis for quantification.

### 2.7 RNA pull‐down assay

RNA pull‐down assay was performed as previously reported.^16^ In Brief, 1 × 10^7^ cells were lysed on ice for 10 min with 500 μl polysome extraction buffer (100 mM KCl, 5 mM Mgcl_2_, 5% NP40 and 20 mM Tris‐HCl [pH 7.4]). The supernatant was collected and incubated with 1–2 μg biotin‐labelled probes at room temperature (RT) for 30 min to form an RNA‐protein complex. The incubating pre‐treated Streptavidin Magnetic beads (BioLabs, S1420S) were added to the reaction mix at RT for 30 min. After six washed with RNA washing buffer, the precipitated RNA‐protein mixture was subjected to western blot analysis. The dot blot assay was carried out to indicate the sense and anti‐sense biotinylated lncRNA probes. Briefly, 300‐ng biotinylated RNA probes were dropped to Amersham Hybond‐N+membrane (GE Healthcare, UK) and cross‐linked by ultraviolet irradiation. Then, the membrane was incubated with .02% methylene blue (internal control) and incubated with anti‐biotin, HRP‐linked antibody (CST, 7075) overnight at 4°C. The membrane was observed using Amersham Imager 680 (GE Healthcare, USA).

### 2.8 Western blotting and co‐immunoprecipitation

Cells were washed once by phosphate buffered saline (PBS) and lysed in radio immunoprecipitation assay (RIPA) buffer containing protease inhibitors. The protein concentrations were quantified with the bicinchoninic acid (BCA) assay reagent (Fdbio Science, CA). For co‐immunoprecipitation (co‐IP) assays, 500‐μg whole cell lysate protein was reacted with primary antibodies (10‐30 μl) overnight at 4 C°and incubated with protein A/G beads at the next day for 4 h at 4C°. Then immunoprecipitated proteins were eluted from the beads for western blotted with indicated antibodies.^13^

### 2.9 Protein half‐life assay

Cells were treated with siRNAs or plasmids for 48 h and then treated with CHX (100 μg/ml), which blocked new protein synthesis. After CHX treatment, cells were collected at indicated times. Western blotting (WB) detected the protein levels.

### 2.10 In vivo ubiquitination assays

His‐Ub and the desired plasmids were transfected into 293T cells. After a 36 h‐transfection, cells were treated with 20‐μM MG‐132 for 6 h and lysed in lysis buffer (0.1 M Na_2_HPO_4_/NaH_2_PO_4_, 10 mM β‐mercaptoethanol, 6 M guanidine‐HCl, 5 mM imidazole and .01 M Tris‐HCl[pH 8.0]). The lysates were treated with sonication and incubated with nickel‐nitrilotriacetic acid beads (QIAGEN) for 4 h at RT. Then, the beads were washed once with buffer A (0.1 M Na_2_HPO_4_/NaH_2_PO_4_, 8 M Urea, 10 mM β‐mercaptoethanol and .01 M Tris‐HCl [pH 8.0]), once with .2%Triton X‐100 + buffer B (8 M Urea, .1 M Na2HPO4/NaH2PO4, .01 M Tris‐HCl and 10 mM β‐mercaptoethanol[pH 6.8]) and one time with .1%Triton X‐100 + buffer B. The proteins were eluted from the beads for WB.^14^

### 2.11 RNA stability assays

Cells were transfected with plasmids or siRNAs for 48 h, then treated with ActD (5 μg/ml) for indicated hours and collected. Total RNA was obtained by the Trizol reagent (Invitrogen, USA) and detected by qRT‐PCR. The degradation rate (*K*_decay_) and half‐life of mRNA (*t*_1/2_) were estimated using the equation: *t*_1/2_*__* = ln2/*K*_decay_ as previously published.^17^

### 2.12 Mammosphere formation assay

Note that 1 × 10^4^ indicated cells were suspended in a 500‐μl DMEM‐F12 serum‐free medium (Invitrogen, USA) and mixed with a 500‐μl Matrigel matrix solution (Corning Life Science, USA). Then, the mixture was gently added on a 6‐well ultra‐low attachment culture plate and placed at 37°C for 4 h to solidify the gel. Four hours later, 2‐ml DMEM‐F12 medium (comprising basic fibroblast growth factor [b‐FGF, 20 ng/ml], epidermal growth factor [20 ng/ml], insulin [5 μg/ml], 4% BSA and 2% B27) was added to the top of gels. Cells were cultured in a CO_2_ incubator for 14 days, and the spheroids were imaged using a 10× microscope (Carl Zeiss, Germany). Measurements were performed in ImageJ software, where the minimized spheroid size was 40 cells per case. Mean values of triplicate replicates were used for data analysis.

### 2.13 Cell viability and apoptosis assay

For cell viability assay, 5000 cells were seeded in a 96‐well plate overnight and treated with inhibitors or drugs as indicated times and concentration. Then, those cells were incubated with MTS dye (Promega, G111A) for 30–60 min and measured at OD 490 nm with the BioTek Gen5 system (BioTeck, USA). The optical density (OD) value was analysed by GraphPad Prism8.0 using non‐linear regression. The equation log (inhibitor) versus response‐variable slope was applied to plot the fitted dose‐response curve. The IC_50_ was calculated as: Y = Bottom + (TopBottom)/(1 + 10ˆ ((Log IC_50_‐X) *HillSlope)).

For apoptosis analysis, 1 × 10^6^ cells were washed with cold PBS and then suspended in binding Buffer. Annexin V‐FITC‐PI dual‐staining kit (BD Biosciences, 556547) was added 1:1 in suspensions and stained at RT for 15‐min photophobic. Stained cells were screened by flow cytometry immediately. Cells were also harvested and using cleaved‐caspase 3 and PARP1 specific antibodies measured by WB.

### 2.14 Xenograft mice experiment

Animal care and experiments were executed following the Institutional Animal Care and Use Committee and National Institute of Health (NIH) guidelines as previously described.^13^ Note that 7 × 10^6^ SGC7901 cells stably overexpressed LNC942 or pCDH empty vector (LNC942/NC) were suspended in 125‐μl PBS and inoculated into the right dorsal flank of 5‐week‐old female nude mice (*n* = 5 per group). After 1 week, the mice were randomly allocated to six groups and treated with DDP (2 or 4 mg/kg), FK228 (1 mg/kg), DDP (2 or 4 mg/kg) combined with FK228 (1 mg/kg) or vehicle PBS via intraperitoneal injection for indicated days (Figure 8A). The tumour volume's widest vertical width and longest diameter were measured before every injection day using a calliper. The tumour volume = length × width^2^ × .5. Finally, the tumour tissues from the xenograft mice were surgically removed, weighed and measured. The obtained tumour tissues were treated with immunohistochemistry staining to detect the apoptosis rate using anti‐cleaved‐caspase 3.

### 2.15 Molecular docking analysis

The LeDock program was implemented to predict the binding mode of FK228 in the binding site of MSI2.^18^ The crystal coordinates for human MSI2 (Protein Data Bank [PDB] code: 6DBP) were retrieved from the PDB database.^19^ The LePro module in the LeDock program was performed to prepare the protein structure of MSI2, including removing water molecules and generating a grid file. Afterward, the LeDock module in the LeDock program was employed to generate 200 conformations.

### 2.16 MSI2 and FK228 binding kinetics analysed by surface plasmon resonance

The binding kinetics of MSI2 fusion protein to MSI2 inhibitor FK228 were performed by the Protein Preparation and Identification Facility, Technology Center for Protein Science, Tsinghua University using surface plasmon resonance (SPR) (Biacore 8K plus, Cytiva). Briefly, recombinant MSI2 protein (Proteintech) was covalently immobilized to a CM5 sensor chip through amine groups in a sodium acetate buffer (pH 4.5) at a final response unit (RU) of about 11545. Then, serial dilutions of FK228 ran through the sensor chip system. The running buffer consisted of HBST (HEPES buffered saline + Tween) buffer and 5% DMSO. The results fit a 1:1 binding model with Biacore Evaluation Software (GE Healthcare).

### 2.17 Human proteome microarray analysis

The HuProtTM human proteome microarray (Wayen Biotechnologies, Shanghai) was used to identify potent LNC942 binding proteins.^20^ Briefly, proteome microarrays were blocked with a pre‐cold SuperBlock T20 Blocking Buffer (Thermo Scientific, USA) for 2 h with gentle agitation. The microarrays were washed with TBST (Tis buffered saline + Tween) and incubated with biotin‐labelled anti‐sense (control) or sense LNC942 probes for 1 h at RT. After being washed in TBST, the dried microarrays were scanned with a GenePix 4000B microarray scanner (Axon Instruments, USA) to evaluate the results. Both probes corresponding to a protein were required to have a z‐score of ⩾3 in two repeated array experiments was considered a positive interaction, resulting in 209 positive hits.

### 2.18 Bioinformatics analysis

The LNC942 or MSI2 knock‐down gene expression profiles were analysed with the gene set enrichment analysis (GSEA) 3.0 software (http://www.broadinstitute.org/gsea/). Kaplan–Meier analysis of the progression‐free and overall survival was conducted according to LNC942 expression from The Cancer Genome Atlas (TCGA) RNA‐seqV3 data of STAD (https://portal.gdc.cancer.gov/).

### 2.19 Statistical analyses

All statistical analyses were executed with GraphPad Prism 8.0 (GraphPad Software, Inc., La Jolla, CA). The difference between the two or more groups was calculated by unpaired Student's *t*‐test or one‐way analysis of variance (ANOVA). The Kaplan–Meier survival plots were analysed using the log‐rank test. The correlation between MSI2 and c‐Myc level in GC tissue was analysed using the chi‐square test. Data were represented as mean ± standard deviation (SD) from at least three independent experiments in vitro and mean ± standard error of mean (SEM) in vivo. The *p*‐value in the figure legends is shown as: no significant (ns), *p* > .05; *p < .05; **p < .01; ***p < .001.

## 3 RESULTS

### 3.1 LNC942 is a new chemoresistance‐related lncRNA that is correlated with poor prognosis in GC

**FIGURE 1:**
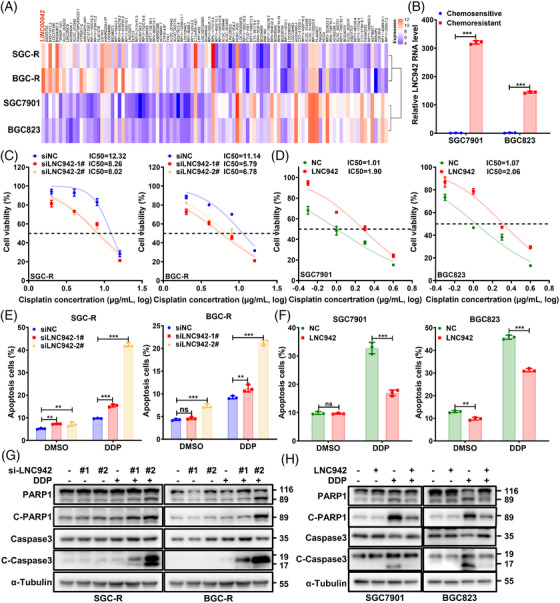
LNC942 promotes gastric cancer (GC) cell resistance to cisplatin in vitro. (A) The heatmap from microarray analysis suggested that LNC942 is the most differentially up‐regulated lncRNA in chemoresistant cells. (B) Quantitative real‐time PCR (qRT‐PCR) detection of the LNC942 expression in chemosensitive and resistant SGC7901 and BGC823 cells. (C) Relative cell viability of SGC‐R and BGC‐R cells with LNC942 siRNA knock‐down (si‐LNC942) compared with that of control cells (si‐NC) treated with the indicated concentrations of cisplatin (DDP) for 24 h. (D) SGC7901 and BGC823 cells with stable LNC942 overexpression were treated with DDP at different concentrations for 24 h, and the resultant cell viability was determined through the MTS assay. (E) PI/Annexin V flow cytometry analysis of DDP‐resistant cells transfected with si‐NC or si‐LNC942, followed by treatment with or without DDP (8 μg/ml) for 24 h. DMSO: solvent control of DDP. (F) The apoptosis of sensitive cells with or without LNC942 overexpression in the presence or absence of DDP treatment (1 μg/ml, 24 h) was measured through flow cytometry and then quantified. (G) Apoptotic cells among LNC942 knocked‐down SGC‐R and BGC‐R cells were treated with DDP (8 μg/ml) for 24 h and then analysed by anti‐cleaved‐PARP1 and cleaved‐caspase 3 through western blotting. (H) Western blotting of SGC7901 and BGC823 cells that were stably expressing LNC942 treated with or without DDP (1 μg/ml, 24 h) with cleaved‐PARP1 and cleaved‐caspase 3 antibody. Data in (B‐F) are represented as mean ± SD of three independent experiments; the *p* value was determined by a two‐tailed unpaired Student's *t* test. ns, *p* > .05; *p < .05; **p < .01; ***p < .001

Chemoresistance is the leading cause of treatment failure among cancer patients. To explore chemoresistance‐related lncRNAs involved in GC, we employed lncRNA microarrays to examine the lncRNA expression profiles of chemosensitive cells (SGC7901 and BGC823) and paired resistant cells (SGC‐R and BGC‐R).^11^ The top 100 differentially expressed lncRNAs are depicted in the heat map, where LNC942 is the most significantly up‐regulated lncRNA in chemoresistant cells (Figure 1A, Table S4). Besides, qRT‐PCR showed a notable up‐regulation of LNC942 in chemoresistant cells (Figure 1B). Moreover, LNC942 was reported to improve the proliferation and progression of breast cancer cells,^21^ albeit its role in the chemoresistance of GC remains unknown. To assess the potential role of LNC942 in chemoresistance, we first knocked down the LNC942 expression in chemoresistant SGC‐R and BGC‐R cells and overexpressed LNC942 in chemosensitive SGC7901 and BGC823 cells. As expected, the expression of LNC942 was either remarkably decreased or increased (Figure S1A,B). Next, we exposed cells to indicate DDP concentrations for 24 h. The cell viability assay indicated that LNC942 depletion restored SGC‐R and BGC‐R cells' sensitivity to DDP and decreased the IC_50_ value relative to those of the control cells (Figure 1C). In contrast, the LNC942 overexpression promoted DDP resistance in SGC7901 and BGC823 cells (Figure 1D). However, LNC942 did not promote the proliferation of chemoresistant GC cells (Figure S1C). Of note, after DDP treatment, the chemoresistant cells transfected with LNC942 siRNAs exhibited increasing signs of apoptosis in PI/Annexin V apoptosis assays (Figure 1E, Figure S1D). In contrast, the LNC942 overexpression in SGC7901 and BGC823 cells strikingly reduced the number of apoptotic cells (Figure 1F, Figure S1E). Besides, WB revealed that the levels of PARP1 and caspase 3 cleavage (cleaved‐PARP1 and cleaved‐caspase 3) were higher in LNC942 knocked‐down cells but lower in LNC942 overexpressing cells compared with those in the control cells (Figure 1G,H). Therefore, LNC942 promotes chemoresistance of GC cells mainly by reducing cell apoptosis instead of affecting cell proliferation.

In order to further understand the clinical significance of LNC942 expression in GC, we analysed the RNA‐seq data from TCGA. The Kaplan–Meier survival curve demonstrated a close correlation between the high level of LNC942 expression and poor overall and progression‐free survival of patients with GC (Figure S1F,G). Taken together, these data indicated that a high level of LNC942 promotes cell resistance to DDP in vitro, highlighting the clinical significance of LNC942 in GC.

### 3.2 LNC942 is directly associated with MSI2

**FIGURE 2:**
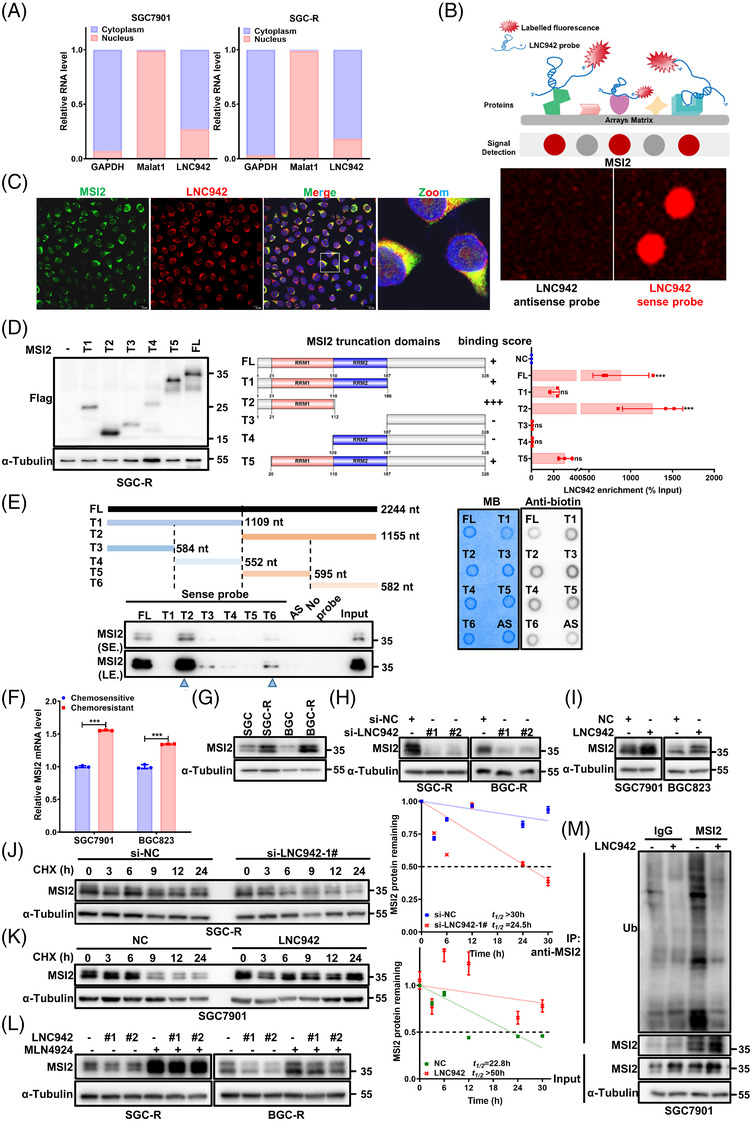
LNC942 interacts with MSI2 protein and prevents its ubiquitination. (A) Quantitative real‐time PCR (qRT‐PCR) detection of LNC942 expression in the cytoplasmic and nuclear fractions. GAPDH is the cytoplasmic marker, and Marat1 is the nuclear marker. (B) Identification of LNC942‐binding protein by human proteome microarray experiment. Upper: Schematic of the human proteome microarray experiment. Bottom: Protein spot subarray demonstrating strand‐specific binding of LNC942 sense strand to MSI2 protein. (C) RNA fluorescence in situ hybridization (FISH) and immunofluorescence (IF) assays showing the co‐localization of LNC942 (Cy3‐labelled, red) and MSI2 (Alexa488‐labelled, green) in SGC‐R cells. Blue: DAPI. All scale bars: 10 μm. (D) RNA immunoprecipitation (RIP) assays were performed with anti‐Flag antibody in SGC‐R cell transfected with vectors expressing Flag‐tagged full length (FL) or truncation mutants of MSI2. Left: Western blotting indicating the expression of Flag‐tagged FL or truncation mutants of MSI2. Middle: Schematic structures of MSI2 proteins and five truncated mutants of MSI2 variants. The binding score indicated the binding intensity between LNC942 and the domains of MSI2. Right: qRT‐PCR detection of LNC942 enrichment after RIP assays. (E) Deletion mapping of the MSI2‐binding region(s) in LINC00942. Left: Western blotting detecting the MSI2 enrichment after RNA pull‐down. AS: anti‐sense probe. Right: the biotin‐labelled LNC942 probes were quantified (300 ng) and detected by the dot blot assay. MB: methylene blue, used as internal reference. (F and G) qRT‐PCR (F) and western blotting (G) were performed for the detection of MSI2 expression in chemosensitive or resistant SGC7901 and BGC823 cells. (H) The protein levels of MSI2 in chemoresistant cells transfected with si‐NC or LNC942 siRNAs. (I) The MSI2 protein expression in SGC7901 and BGC823 cells stably expressing LNC942. (J) SGC‐R cells with or without LNC942 knock‐down were treated with cycloheximide (CHX) (100 μg/ml) for the indicated time points. MSI2 protein abundance was analysed through western blotting, followed by quantification using ImageJ. (K) Western blotting detection of MSI2 protein half‐life in LNC942 stably expressed SGC7901 cells with CHX (100 μg/ml) for the indicated time points. MSI2 protein abundance was quantified using ImageJ. (L) Western blotting of MSI2 in SGC‐R or BGC‐R cells transfected with or without si‐LNC942 following proteasome inhibition with MLN4924 (1 μM, 24 h). (M) SGC7901 cells stably expressing LNC942 were treated with 20 μM MG‐132 for 6 h and then subjected to IP assays to detect the MSI2 ubiquitination levels. Data in panels (A), (D), (F), (J) and (K) are represented as mean ± SD of the three independent experiments. The *p* value was determined using a two‐tailed unpaired Student's *t* test. ns, *p* > .05; *p < .05; **p < .01; ***p < .001

Cell fractionation assays (Figure 2A) and RNA FISH assay (Figure S2A) revealed that LNC942 is located mainly in the cytoplasm. LncRNAs may be involved in regulating various cellular processes through protein–lncRNA interactions^22^; therefore, we attempted to identify LNC942‐associated proteins through human proteome microarray by using a fluorescence‐labelled LNC942 probe (Figure 2B). The sense LNC942 rather than the anti‐sense control interacted specifically with MSI2 with the highest fluorescence signal (Figure 2B). The interaction between LNC942 and MSI2 was verified using the RIP assay, which displayed a significant enrichment of LNC942 with MSI2 in chemoresistant cells (Figure S2B). Consistently, the RNA pull‐down assay showed that MSI2 specifically binds to the sense LNC942 probe (Figure S2C). IF and RNA FISH assays also indicated that endogenously expressed LNC942 was mainly co‐localized with MSI2 in the cytoplasm (Figure 2C).

As a canonical RBP, MSI2 consists of two highly conserved RNA‐recognition motifs (RRMs), wherein RRM1 plays a crucial role in determining substrate specificity, whereas RRM2 is more or less supportive.^9^ To investigate which domain of MSI2 mediates the LNC942–MSI2 interaction, we performed RIP assays using antibodies against Flag‐tagged full‐length (FL) and truncated MSI2 (Figure 2D). Protein domain mapping revealed that the RRM1 domain of MSI2 must associate with LNC942 (Figure 2D). Besides, to determine the region of LNC942 that binds to MSI2, a series of LNC942 fragments were generated. The results demonstrated that MSI2 mainly binds to fragment 6 (1162‐2244 nt) of the LNC942 (Figure 2E). These results collectively illustrated that LNC942/MSI2 specifically formed an RNA/protein complex through the internal region (1162‐2244 nt) of the LNC942 and the RRM1 domain MSI2 in chemoresistant GC cells.

### 3.3 LNC942 stabilizes MSI2 by preventing SCF^β‐TRCP^ E3 Ub ligase‐mediated ubiquitination

We further evaluated the effect of the LNC942–MSI2 interaction on MSI2. Although MSI2 mRNA and protein levels were higher in resistant cells than in sensitive cells (Figure 2F,G), LNC942 knock‐down or overexpression significantly modified only the protein expression of MSI2 (Figure 2H,I) but not its mRNA level (Figure S2D,E), suggesting that LNC942 regulates the protein levels of MSI2 possibly at the translational level or through post‐translational modifications. We then used CHX to block the synthesis of new proteins and found that MSI2 protein stability was decreased upon LNC942 deletion (Figure 2J, Figure S2F). Conversely, the half‐life of MSI2 protein was extended in chemosensitive cells overexpressing LNC942 compared with that in control cells (Figure 2K, Figure S2G). Previous studies have revealed that MSI2 undergoes constant proteasomal degradation through polyubiquitination.^23^, ^24^ Indeed, the MSI2 protein accumulated after the treatment of cells with the proteasomal inhibitor MG‐132 (Figure S2H). Cullin‐based RING‐type E3 ligase complexes (CRLs) is the most prominent family member of E3 ligases in the human genome. Therefore, a specific NEDD8‐activating enzyme inhibitor MLN4924, which makes all CRLs inoperative through inhibiting Cullin neddylation, was also used to explore the influence on MSI2 abundance in cells^25^ (Figure S2H). In addition, MG‐132 (Figure S2I) or MLN4924 (Figure 2L) prevented the decrease in MSI2 protein level caused by LNC942 deletion. Moreover, LNC942 overexpression significantly reduced the level of ubiquitinated MSI2 (Figure 2M). These results indicated that LNC942–MSI2 binding might confer stability to the MSI2 protein by preventing its ubiquitination.

**FIGURE 3:**
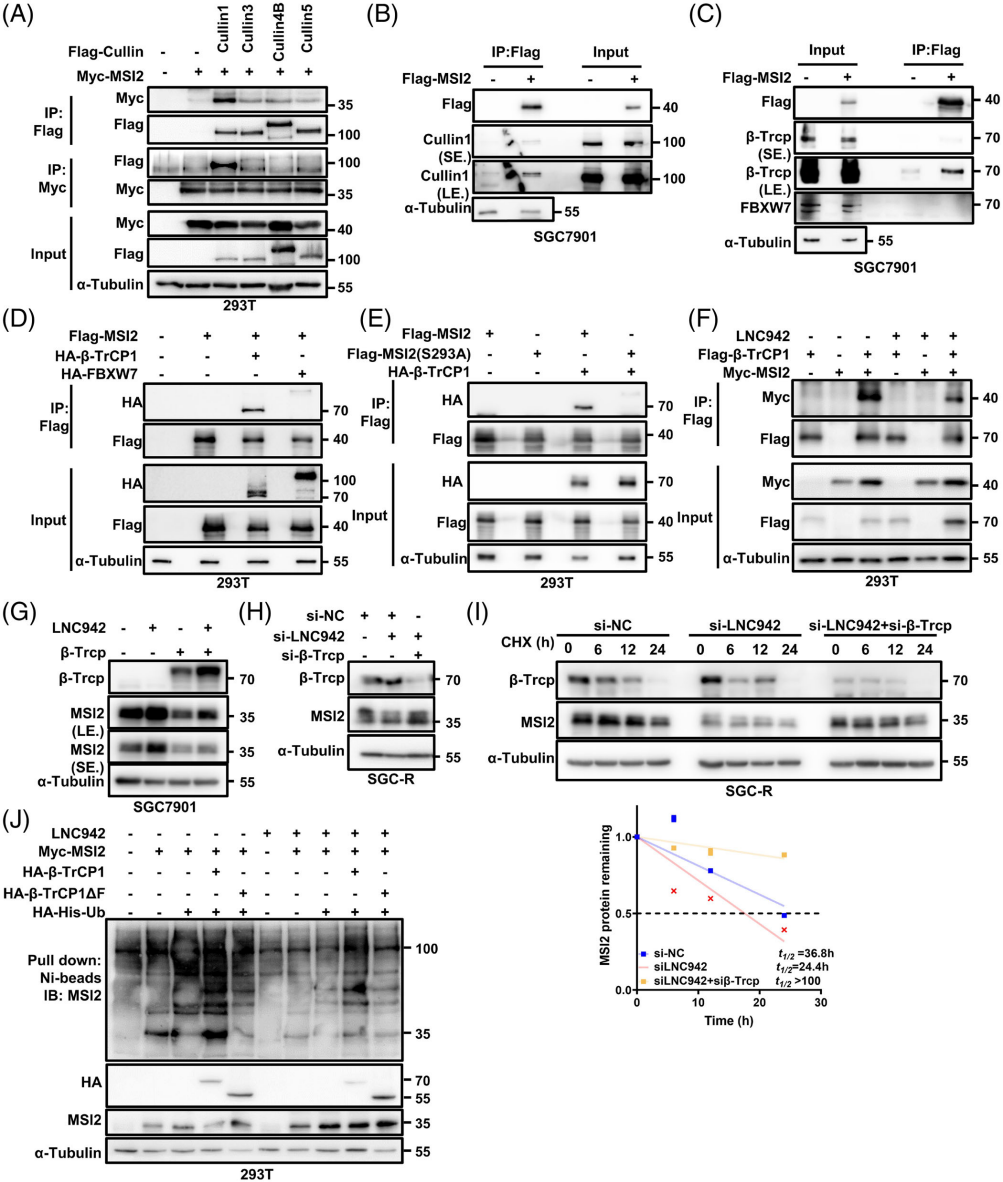
LNC942 disrupts the interactions between MSI2 and the SCF^β‐TRCP^ E3 ubiquitin ligase. (A) 293T cells were transfected with empty vector or Flag–Cullin family proteins and Myc‐MSI2 plasmids as indicated, followed by immunoprecipitation (IP) with anti‐Flag or anti‐Myc antibody and western blotting with the indicated antibodies. α‐Tubulin served as the loading control. After 48 h of transfection, the cells were treated with MG‐132 (20 μM) for 6 h before harvesting. (B) IP with anti‐Flag antibody was performed in SGC7901 cells transfected with empty vector or Flag–MSI2 plasmids. Western blotting was performed with the indicated antibodies. The cells were treated with MG‐132 (20 μM) for 6 h before harvesting. (C) Western blotting of input and anti‐Flag IP derived from SGC7901 cells transfected with Flag‐MSI2 plasmids. The cells were treated with MG‐132 (20 μM) for 6 h before harvesting. (D) Western blotting analysis of input and anti‐Flag IP derived from 293T cells transfected with Flag‐MSI2, HA‐β‐Trcp or HA‐FBXW7 plasmids. The cells were treated with MG‐132 (20 μM) for 6 h before harvesting. (E) Western blotting analysis of input and anti‐Flag IP derived from 293T cells transfected with HA‐β‐Trcp and the indicated Flag‐tagged MSI2 constructs. The cells were treated with MG‐132 (20 μM) for 6 h before harvesting. (F) IP was performed with anti‐Flag antibody in 293T cells transfected with Flag‐β‐Trcp and Myc‐MSI2 plasmids in the presence or absence of LNC942, followed by western blotting with the indicated antibodies. The cells were treated with MG‐132 (20 μM) for 6 h before harvesting. (G) The SGC7901 cells were co‐transfected with LNC942 and β‐Trcp plasmids, followed by western blotting of the MSI2 and β‐Trcp expression. (H) The SGC‐R cells were co‐transfected with LNC942 and β‐Trcp siRNAs, followed by western blotting of the MSI2 and β‐Trcp expression. (I) Western blotting detection of MSI2 protein half‐life in SGC‐R cells transfected with the indicated siRNAs and treated with cycloheximide (CHX) (100 μg/ml). The MSI2 protein abundance was quantified using ImageJ in the bottom panel. (J) 293T cells were co‐transfected with the indicated plasmids and treated with MG‐132 (20 μM) for 6 h before collection. MSI2 pull‐down experiments were conducted with nickel‐nitrilotriacetic acid (Ni‐NTA) beads, and the samples were analysed using western blotting with the indicated antibodies. Ub, ubiquitin. Data in (I) are shown as mean ± SD of three independent experiments

Either MLN4924 or MG‐132 treatment could increase endogenous MSI2 protein abundance in sensitive cells (Figure S2H), suggesting certain unidentified CRLs may negatively regulate the MSI2 stability in GC cells. We then employed a Cullin family protein panel (Cullin 1, 3, 4B, and 5) to identify the potential E3 Ub ligase involved in LNC942‐mediated MSI2 stabilization. As shown in Figure 3A, Cullin1, rather than other Cullin family members (such as Cullin 3, 4B and 5), significantly interacted with MSI2. Furthermore, co‐IP experiments indicated the interaction of exogenously expressed MSI2 with endogenous Cullin1 (Figure 3B). Moreover, the deletion of Cullin1 dramatically increased the MSI2 protein level in SGC7901 and BGC823 cells, suggesting that Cullin1‐based CRL plays a vital role in governing MSI2 stability in GC cells (Figure S3A). Moreover, silencing LNC942 prevented the increase in MSI2 protein level caused by Cullin1 depletion (Figure S3B). Conversely, Cullin1‐dependent degradation of MSI2 could be hindered by the ectopic overexpression of LNC942 (Figure S3C). These results suggested that LNC942 is involved in preventing Cullin1‐dependent degradation of MSI2.

CRL1, or F‐box protein–CUL1–S‐phase kinase‐associated protein 1 complex, employs variable F‐box proteins as substrate adaptors to confer substrate specificity.^26^ Interestingly, a potential conserved DSGXX degron motif for β‐TrCP recognition exists in MSI2 proteins in various species (Figure S3D),^27^ suggesting that β‐TrCP possibly catalysed MSI2 ubiquitination. Consistently, MSI2 exclusively interacted with β‐Trcp instead of other CUL1‐based E3 ligase adaptor proteins, such as FBXW7 (Figure 3C,D). The ectopic overexpression of β‐Trcp, rather than FBXW7, markedly decreased the abundance of MSI2 protein in a concentration‐dependent manner (Figure S3E,F). The depletion of β‐Trcp, rather than FBXW7, caused an increase in the level of MSI2 protein (Figure S3G,H). Moreover, MSI2 protein carrying mutations in phosphor‐degron (S293A) failed to bind to β‐Trcp (Figure 3E). Notably, LNC942 was also involved in the degradation of MSI2 by β‐TrCP, as LNC942 could interrupt the interaction of MSI2 with β‐Trcp (Figure 3F) and prevent the decrease in its level caused by β‐TrCP overexpression (Figure 3G). β‐TrCP knock‐down also failed to increase the MSI2 protein level (Figure 3H) and its half‐life (Figure 3I) in SGC‐R cells when LNC942 silencing. More importantly, exogenous wild‐type β‐TrCP but not the enzyme activity‐defective mutant (β‐TrCPΔF) promoted MSI2 ubiquitination, whereas overexpression LNC942 prevented this ubiquitination (Figure 3J). Therefore, we speculate that LNC942 specifically binds to MSI2 to block its interaction with β‐Trcp, thereby inhibiting SCF^β‐TRCP^ E3 Ub‐mediated ubiquitination and the subsequent degradation of MSI2.

### 3.4 LNC942 promotes chemoresistance by targeting MSI2 in vitro

**FIGURE 4:**
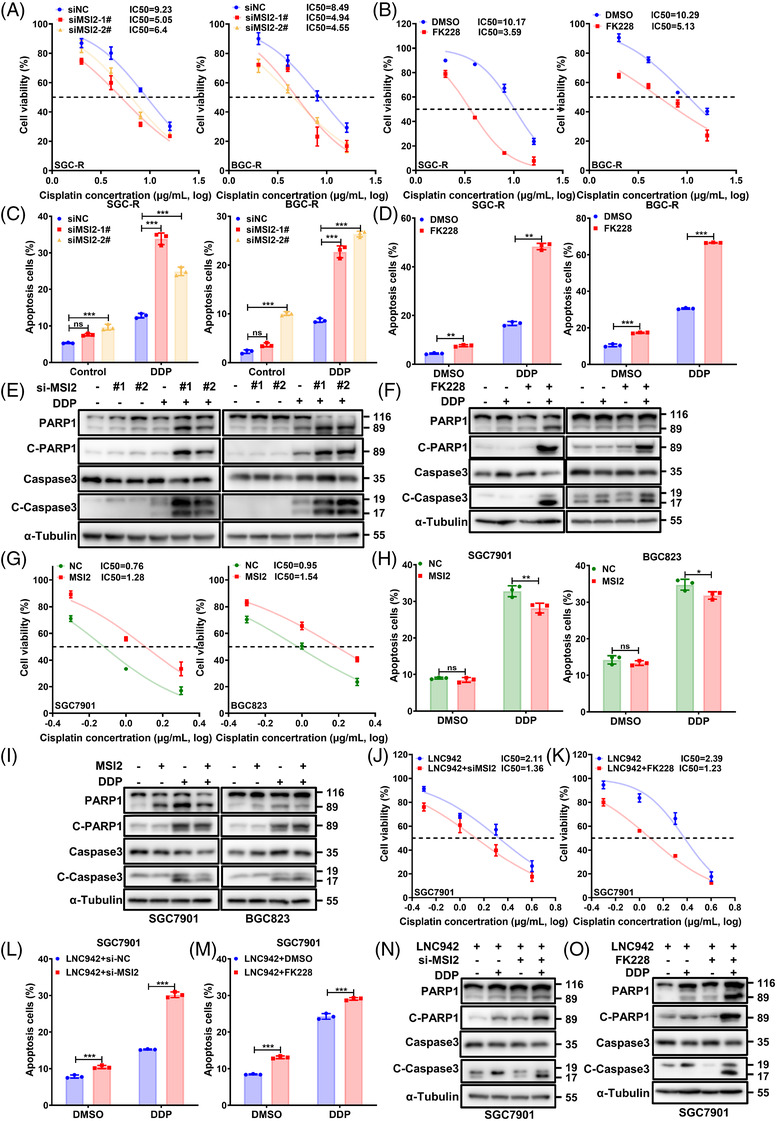
MSI2 promotes gastric cancer (GC) cell resistance to cisplatin in vitro. (A) The SGC‐R and BGC‐R cells were transfected with si‐NC or si‐MSI2 treated with cisplatin (DDP) at different concentrations for 24 h, and the cell viability was then determined using the MTS assay. (B) SGC‐R and BGC‐R cells were treated with MSI2 inhibitor, FK228 (.125 μg/ml) and different concentrations of DDP for 24 h, and the cell viability was then measured using the MTS assay. DMSO: solvent control of FK228. (C) PI/Annexin V staining and flow cytometry in chemoresistant cells following si‐NC or si‐MSI2 transfection and treatment with or without DDP (8 μg/ml) for 24 h. (D) Resistant cells were treated with DDP (8 μg/ml), FK228 (.125 μg/ml) or DDP plus FK228 for 24 h. The apoptosis was measured by flow cytometry, and the number of apoptosis cells was quantified. (E) Apoptotic cells among MSI2 knocked‐down SGC‐R and BGC‐R cells treated with DDP (8 μg/ml) for 24 h were measured using anti‐cleaved‐PARP1 and cleaved‐caspase 3 through western blotting. (F) The caspase 3 and PARP1‐cleaved activities of resistant cells treated with DDP (8 μg/ml), FK228 (.125 μg/ml) or their combination for 24 h were analysed through western blotting. (G) The SGC7901 and BGC823 cells with ectopic overexpression of MSI2 were treated with different concentrations of DDP for 24 h, and the resultant cell viability was determined using the MTS assay. (H) Apoptosis of sensitive cells with or without MSI2 overexpression in the presence or absence of DDP treatment (1 μg/ml, 24 h) was evaluated through flow cytometry and then quantified. (I) Western blotting was performed to detect cleaved‐caspase 3 and cleaved‐PARP1 in sensitive cells with MSI2 overexpression following DDP treatment (1 μg/ml, 24 h). (J) Stable SGC7901 cells overexpressing LNC942 were transfected with MSI2 siRNAs and treated with different concentrations of DDP for 24 h, following which the cell viability was measured using the MTS assay. (K) Cell viability of stable SGC7901 cells overexpressing LNC942 treated with FK228 (.125 μg/ml) and different concentrations of DDP for 24 h was measured using the MTS assay. (L and N) SGC7901 cells that were stably overexpressing LNC942 were transfected with MSI2 siRNAs for 48 h and then treated with DDP (1 μg/ml) for 24 h, after which the apoptotic cells were analysed through flow cytometry (L) and western blotting (N). (M and O) SGC7901 cells stably overexpressing LNC942 were co‐treated with FK228 (.125 μg/ml) and DDP (1 μg/ml) for 24 h. Apoptotic cells were analysed by flow cytometry (M) and western blotting (O). Data in (A‐D), (G), (H), (J‐M) are represented as mean ± SD of the three independent experiments; the *p* value was determined using a two‐tailed unpaired Student's *t* test. ns, *p* > .05; *p < .05; **p < .01; ***p < .001

We further explored the biological role of MSI2 in GC chemoresistance, considering its high expression in chemoresistant cells (Figure 2F,G). We first measured the resistant cells’ viability treated with DDP at different concentrations for 24 h. Either knocking down of MSI2 with siRNAs (Figure 4A) or using chemical MSI2 inhibitor FK228 (Figure 4B), resulted in a lower IC_50_ value than that in the control group (scramble siRNAs or DMSO). In line with this result, MSI2 silencing increased the proportion of apoptotic cells when chemoresistant cells were treated with DDP (Figure 4C,D, Figure S4H,I). Additionally, the level of cleaved‐PARP1 and cleaved‐caspase 3 remarkably increased when MSI2 was silenced (Figure 4E,F). On the contrary, MSI2 overexpression promoted DDP resistance (Figure 4G) and reduced apoptosis of SGC7901 and BGC823 cells (Figure 4H,I, Figure S4J). Notably, the effect of MSI2 knock‐down on GC sensitivity to DDP could be rescued by overexpressing LNC942 (Figure 4J,K). Moreover, compared with LNC942 overexpression alone, the inhibition of MSI2 increased cell apoptosis upon DDP treatment (Figure 4L‐O, Figure S4K). Consistent with the role of LNC942 (Figure S1C), inhibiting MSI2 did not affect the proliferation of resistant GC cells either (Figure S4F,G). Based on these results, we inferred that LNC942 induces chemoresistance by targeting MSI2.

Notably, we identified FK228, a new small‐molecule antagonist for MSI2 targeting. FK228 is a histone deacetylase inhibitor that possesses anti‐tumour activities,^28^, ^29^ and the US Food and Drug Administration approved drug for treating cutaneous and peripheral T‐cell lymphoma.^30^, ^31^ In this study, we executed molecular docking to predict the capacity of FK228 to bind with MSI2 protein (Figure S4A). SPR^32^ was carried out to verify the direct binding between FK228 and MSI2 (Figure S4B). Besides, FK228 inhibition on cell viability was confirmed by MTS in chemoresistant cells (IC_50_ at around .14 μg/ml for SCG‐R cells and .19 μg/ml for BCG‐R cells) (Figure S4C). Moreover, WB confirmed that FK228 could markedly decrease the protein levels of MSI2 (Figure S4D) and the RNA binding capability of MSI2 (Figure S4E) at the concentration of .125 μg/ml (∼IC_50_). More importantly, FK288 treatment was found to function similar to MSI2 siRNAs in reversing chemoresistance in GC cells (Figure 4B,D,F,K,M,O). These results indicated that FK228 could be a promising MSI2 inhibitor for overcoming chemoresistance in GC.

### 3.5 LNC942 stabilizes *c‐Myc* mRNA through MSI2

**FIGURE 5:**
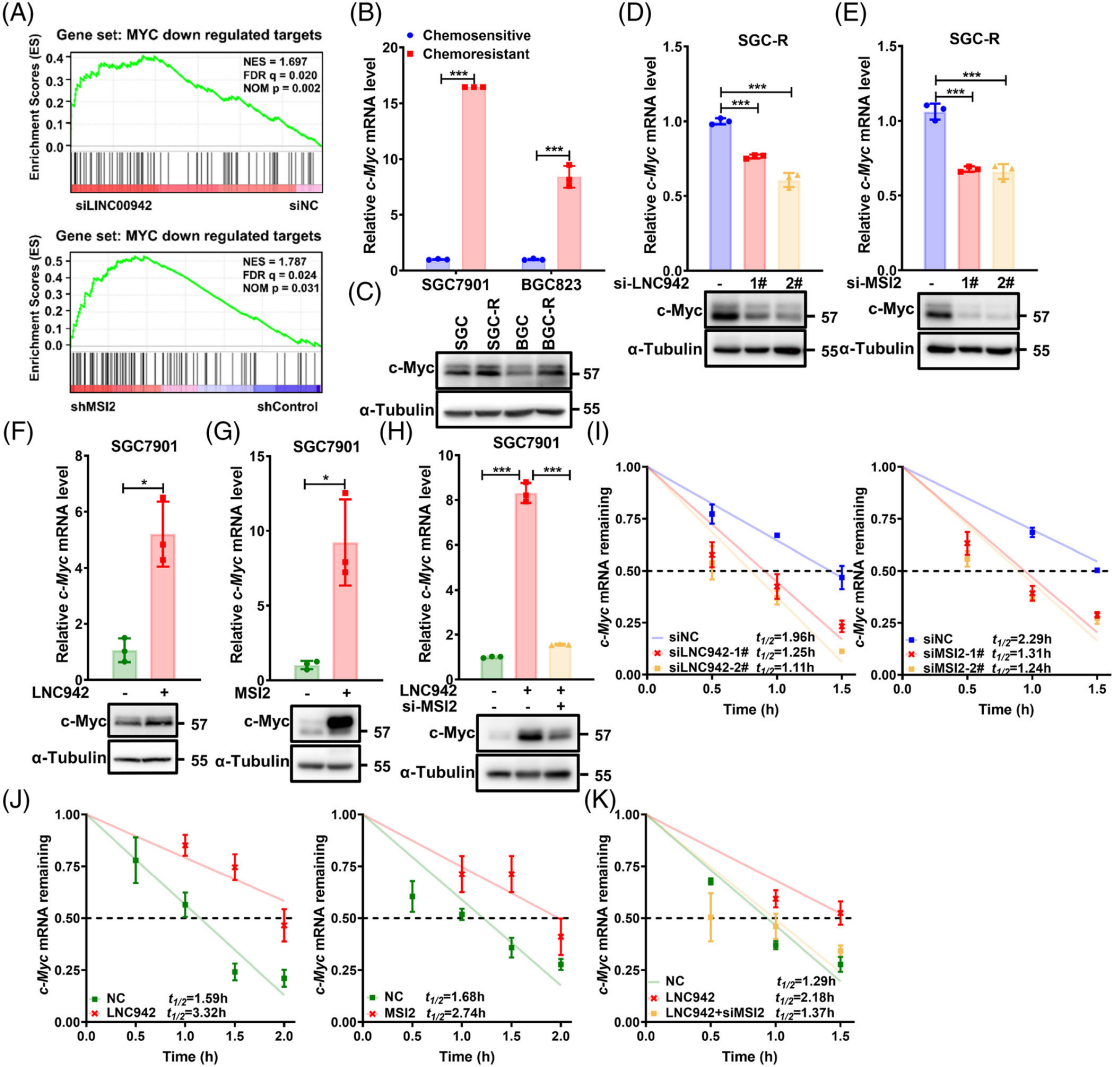
LNC942 increases MSI2 downstream c‐Myc expression. (A) Gene set enrichment analysis (GSEA) of MYC target gene sets in the expression profiles of SGC‐R cells expressing LNC942 siRNA or shMSI2 expression profiles from the GEO database (GSE70685). (B and C) The *c*‐*Myc* mRNA and c‐Myc protein levels in resistant and sensitive cells were determined through quantitative real‐time PCR (qRT‐PCR) (B) and western blotting (C), respectively. (D and E) qRT‐PCR (top panel) and western blotting (bottom panel) analyses of *c*‐*Myc* mRNA and c‐Myc protein expression in SGC‐R cells transfected with LNC942 (D) or MSI2 (E) siRNAs. (F) qRT‐PCR (top panel) and western blotting (bottom panel) analyses of *c*‐*Myc* mRNA and c‐Myc protein expressions in SGC7901 cells stably expressing LNC942. (G) qRT‐PCR (top panel) and western blotting (bottom panel) analyses of *c*‐*Myc* mRNA and c‐Myc protein expressions in SGC7901 cells transfected with MSI2 plasmid. (H) qRT‐PCR (top panel) and western blotting (bottom panel) analyses of *c*‐*Myc* mRNA and c‐Myc protein expression in SGC7901 cells stably expressing LNC942 and transfected with MSI2 siRNA. (I) qRT‐PCR analysis of the decay rate of *c*‐*Myc* mRNA upon LNC942 (left panel) or MSI2 (right panel) inhibition in SGC‐R cells. (J) LNC942 (left panel) or MSI2 (right panel) overexpression enhanced the half‐life of *c*‐*Myc* mRNA. (K) The enhancement of the *c*‐*Myc* mRNA half‐life in SGC7901 cells induced by LNC942 was blocked by silencing MSI2. Data in (B) and (D‐K) are represented as mean ± SD of the three independent experiments; the *p* value was determined using a two‐tailed unpaired Student's *t* test. ns, *p* > .05; *p < .05; **p < .01; ***p < .001

MSI2 overexpression has been reported to cause an increase in the expression of gene signature characteristics of the MYC pathways and control its translation through direct binding.^33^, ^34^ GSEA revealed that several of the predicted targets of MYC were enriched in gene sets that were down‐regulated by LNC942 or MSI2 depletion (Figure 5A). In line with the oncogenic role of c‐Myc, its mRNA and protein levels were markedly up‐regulated in resistant cells than those in sensitive cells (Figure 5B,C). Importantly, the overexpression of LNC942 or MSI2 dramatically elevated the *c*‐*Myc* mRNA and c‐Myc protein expression (Figure 5F,G, Figure S5C‐D). In contrast, the knock‐down of LNC942 or MSI2 was found to have contrasting effects (Figure 5D,E, Figure S5A‐B). Silencing LNC942 or MSI2 also resulted in the significant down‐regulation of c‐Myc downstream genes enriched by GSEA, which confirms the essential role of LNC942 and MSI2 in sustaining c‐Myc functions (Figure S5E). Furthermore, RNA stability assays indicated that LNC942 or MSI2 knock‐down in SGC‐R cells leads to a declined half‐life of *c‐Myc* mRNA (Figure 5I). Conversely, the overexpression of LNC942 or MSI2 increased the *c‐Myc* stability (Figure 5J). Moreover, MSI2 silencing could partially reverse the increase in *c*‐*Myc* mRNA and c‐Myc protein expression (Figure 5H) and half‐life of its mRNA (Figure 5K) after the overexpression of LNC942 in SGC7901 cells. Collectively, these results imply that LNC942 regulates the stability of *c‐Myc* mRNA through MSI2.

### 3.6 MSI2 promotes the stability of *c‐Myc* mRNA in an m^6^A‐dependent manner

**FIGURE 6:**
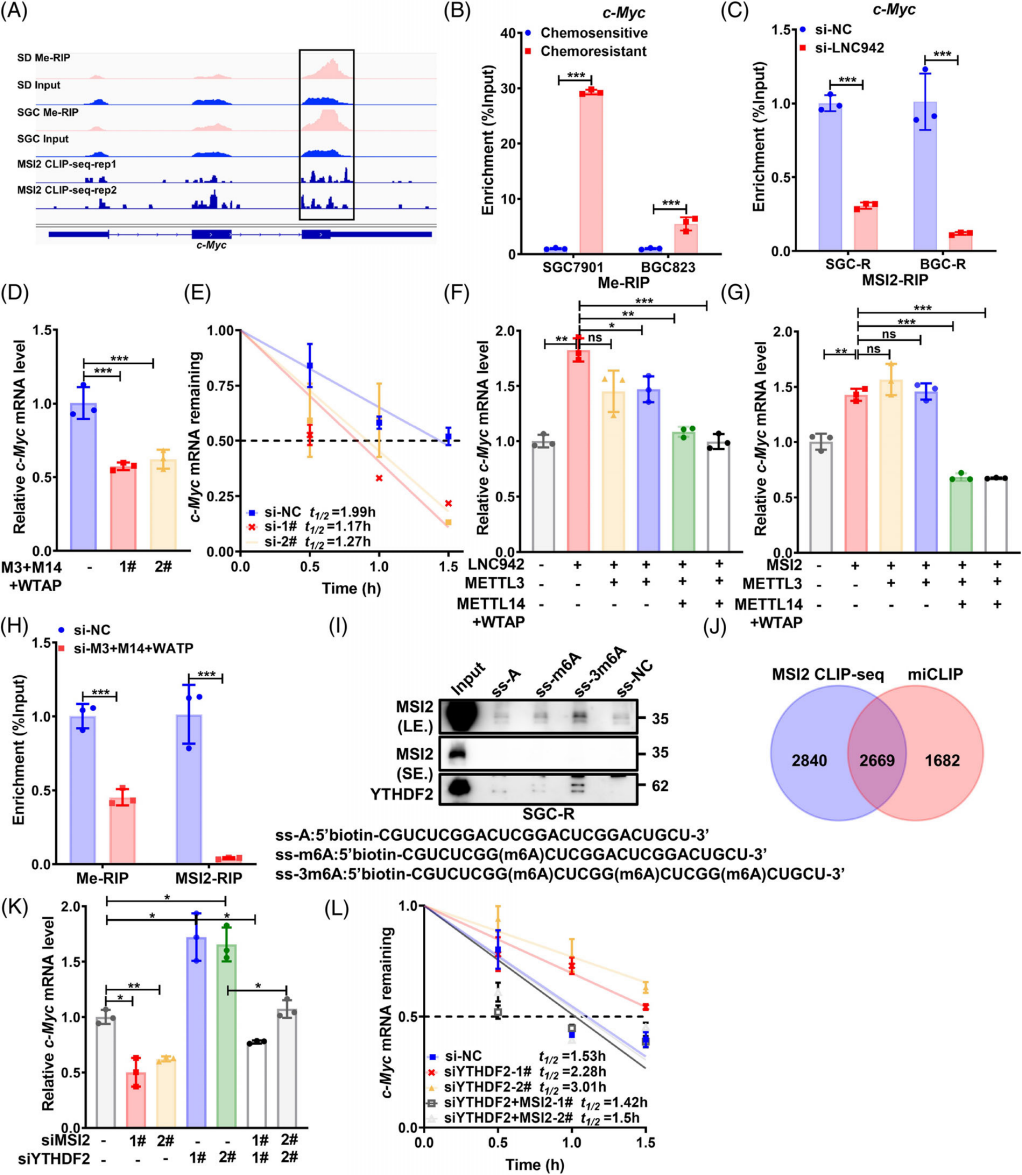
MSI2 promotes the stability of *c*‐*Myc* in an m^6^A‐dependent manner. (A) Integrative genomics viewer tracks displaying Me‐RNA immunoprecipitation (RIP)‐seq and MSI2 CLIP‐seq (GEO: GSE69583) read distributions in *c*‐*Myc* mRNA. Significant peaks are indicated within a box. (B) The detection of m^6^A enrichment in *c*‐*Myc* mRNA in chemosensitive and chemoresistant cells by Me‐RIP and quantitative real‐time PCR (qRT‐PCR). (C) MSI2‐ RIP and qRT‐PCR analysis of the binding between MSI2 and *c*‐*Myc* mRNA with or without LNC942 knock‐down in SGC‐R and BGC‐R cells. (D) The qRT‐PCR detection of the mRNA level of *c*‐*Myc* in SGC‐R cell with or without METTL3‐METTL14‐WTAP complex knock‐down. (E) qRT‐PCR detection of the mRNA half‐life of *c*‐*Myc* after treatment with ActD (5 μg/ml) for the indicated time points. (F and G) LNC942 (F) or MSI2 (G) overexpressing SGC7901 cells were transfected with METTL3 or METTL3+METTL14+WTAP siRNAs, and the mRNA level of *c*‐*Myc* was measured through qRT‐PCR. (H) RIP and qRT‐PCR analysis of the m^6^A modifications of *c*‐*Myc* and the binding of MSI2 to *c*‐*Myc* in METTL3‐METTL14‐WTAP knock‐down or control SGC‐R cells. (I) Western blotting revealed that MSI2 and YTHDF2 (positive control) were pulled down with m^6^A‐containing RNA probes (ss‐m^6^A or ss‐3m^6^A) in SGC‐R cells. (J) Venn diagram illustrating the overlap of MSI2‐binding targets (GEO: GSE69583) and m^6^A‐contained genes (GEO: GSE98623). (K and L) Changes in the *c*‐*Myc* mRNA levels (K) or half‐life (L) in MSI2 or/and YTHDF2 knocked down SGC‐R cells. Data in (B‐H), (K) and (L) panels are represented as the mean ± SD of the three independent experiments; the *p* value was determined by a two‐tailed unpaired Student's *t* test. ns, *p* > .05; *p < .05; **p < .01; ***p < .001

As the most common mRNA modification in eukaryotes, M^6^A is interpreted by its reader proteins to regulate the fate of mRNA.^35^ To elucidate the molecular mechanism of MSI2 in stabilizing *c‐Myc* mRNA and its contribution to chemoresistance, we compared the MSI2 binding RNAs with m^6^A‐modified transcripts that are differentially expressed in the resistant and sensitive cells. The m^6^A‐RIP‐sequencing (Me‐RIP‐seq) and MSI2 crosslinking‐immunoprecipitation sequencing (CLIP‐seq) data^36^ revealed the co‐localization of m^6^A and MSI2‐binding peaks within *c‐Myc* mRNA (Figure 6A). Furthermore, we confirmed that *c‐Myc* mRNA in chemoresistant cells bears more m^6^A modifications than in sensitive cells (Figure 6B). A previous study^21^ reported that LNC942 directly recruits METTL14 protein to stabilize the expression of downstream targets through post‐transcriptional m^6^A modifications. However, LNC942 failed to pull down c‐*Myc* mRNA (Figure S6A); silencing LNC942 up‐regulated the m^6^A level of *c‐Myc* mRNA in GC cells (Figure S6B); silencing METTL14 alone could not decrease the *c‐Myc* mRNA and c‐Myc protein expression (Figure S6C). Moreover, LNC942 facilitated the binding of MSI2 with *c‐Myc* mRNA (Figure 6C). Therefore, we speculate that *c‐Myc* mRNA stability is related to LNC942‐mediated MSI2 recruitment in an m^6^A‐dependent manner instead of directly regulating the m^6^A modifications of *c*‐*Myc* mRNA by LNC942. Indeed, a change in the cellular m^6^A level caused by knocking down of the METTL3‐METTL14–WTAP complex could significantly down‐regulate the expression of *c*‐*Myc* mRNA (Figure 6D) and c‐Myc protein (Figure S6C) as well as impair the *c‐Myc* mRNA stability (Figure 6E). Notably, LNC942 or MSI2 overexpression did not elevate the *c*‐*Myc* mRNA (Figure 6F,G) and c‐Myc protein (Figure S6E,F) levels once the METTL3‐METTL14–WTAP complex was knocked down. Moreover, silencing the METTL3‐METTL14–WTAP complex greatly impaired the binding between *c‐Myc* mRNA and MSI2, highlighting the dependence of *c‐Myc* mRNA–MSI2 interaction on m^6^A modifications (Figure 6H).

*c*‐*Myc* is a well‐documented target of insulin‐like growth factor 2 mRNA binding proteins (IGF2BPs), which stabilized *c‐Myc* mRNAs in an m^6^A‐dependent manner.^37^ Surprisingly, knocking down IGF2BPs had no effect on the *c*‐*Myc* mRNA or protein expression (Figure S6G). Therefore, we speculate that an alternative mechanism(s) is responsible for stabilizing m^6^A‐modified *c‐Myc* mRNA. A previous study indicated that the binding sites of MSI2 in target mRNAs are enriched with a significant m^6^A consensus sequence ʻGGACʼ.^37^ To decipher the relationship between MSI2 and m^6^A modifications, we first performed an RNA pull‐down assay using methylated RNA baits (ss‐m^6^A and ss‐3m^6^A) containing known consensus sites of G (m^6^A) C versus an unmethylated control (ss‐A) (Figure 6I). MSI2 selectively binds to the methylated bait instead of unmethylated control, with a higher affinity to ss‐3m^6^A than to ss‐m^6^A (Figure 6I). Furthermore, we analysed MSI2 CLIP‐seq^36^ and m^6^A individual‐nucleotide‐resolution CLIP (miCLIP) data^38^ to demonstrate that MSI2 binds preferably to m^6^A‐containing RNA (Figure 6J). In addition, 48% (2669/5509) of the peaks bound by MSI2 were found to overlap with m^6^A peaks, which indicated the enrichment of m^6^A modifications at the MSI2 binding regions (Figure 6J). Taken together, MSI2 might be a potential m^6^A binding protein that can stabilize *c‐Myc* mRNA with m^6^A modifications.

*c*‐*Myc* is also a well‐known target of YT521‐B homology domain‐containing family protein 2 (YTHDF2), which is a primary m^6^A reader in charge of mRNA decay.^39^ Indeed, the *c*‐*Myc* mRNA (Figure 6K) and protein level (Figure S6H) were found to be increased, and its mRNA half‐life (Figure 6L) was found to be extended after the knock‐down of YTHDF2. These events were reversed following the co‐suppression of MSI2 expression (Figure 6K,L, Figure S6H). In conclusion, MSI2 binds to *c‐Myc* mRNA having m^6^A modifications to protect it against YTHDF2‐dependent degradation.

### 3.7 LNC942 promotes chemoresistance by upregulating c‐Myc

**FIGURE 7:**
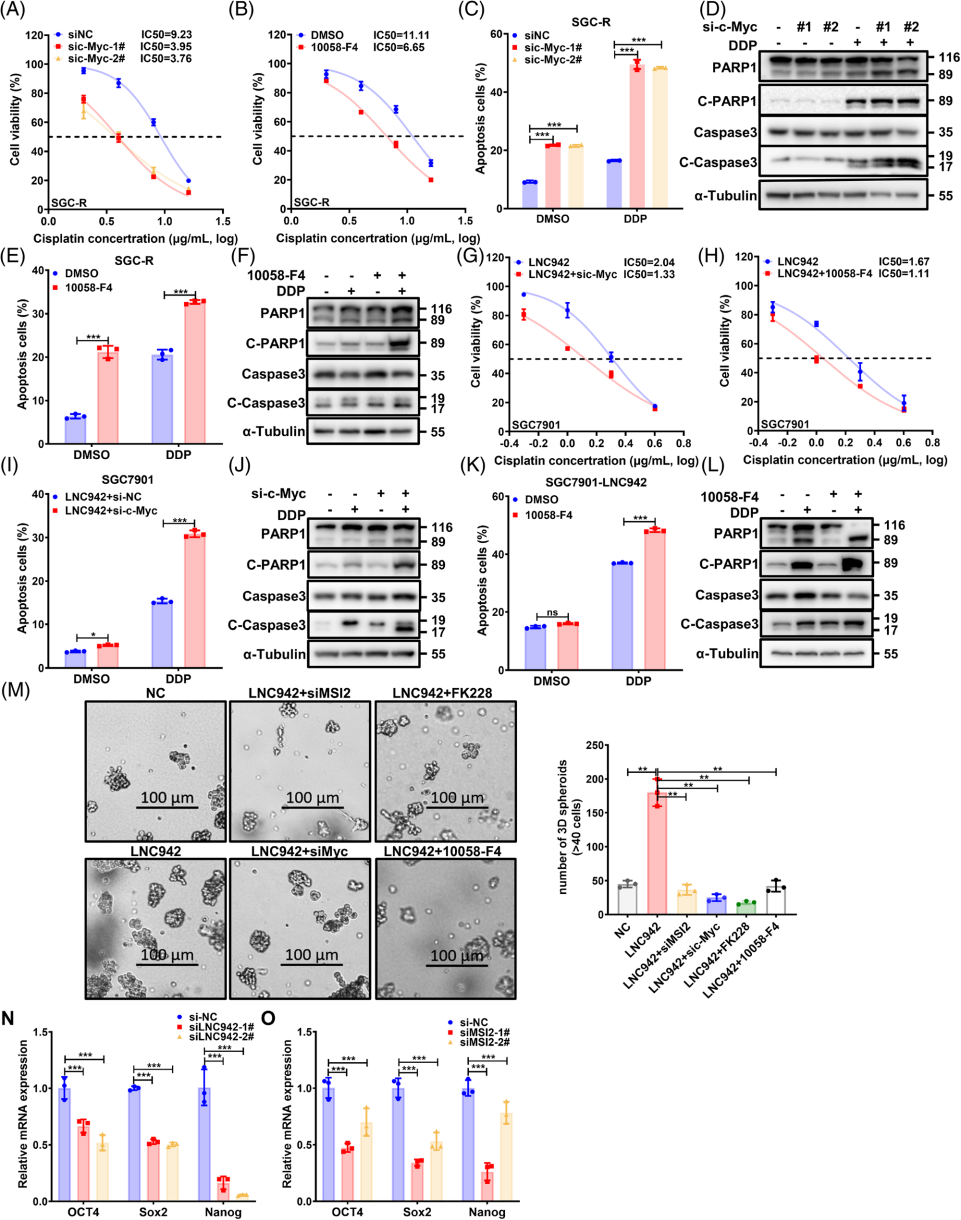
c‐Myc promotes gastric cancer cell resistance to cisplatin in vitro. (A) The assessment of the viability of SGC‐R cells transfected with control or c‐Myc siRNAs with or without cisplatin (DDP) treatment for 24 h by using the MTS assay. (B) The effect of c‐Myc inhibition using the chemical c‐Myc inhibitor 10058‐F4 (6.25 μg/ml) on the viability of resistant cells with or without DDP treatment for 48 h, as detected by the MTS assay. (C and D) SGC‐R cells transfected with control siRNA (si‐NC) or c‐Myc siRNAs were treated with or without DDP (8 μg/ml) for 24 h, and the apoptosis level was measured by flow cytometry (C) as well as western blotting (D). (E and F) SGC‐R cells were pre‐treated with 10058‐F4 (6.25 μg/ml) for 24 h, followed by treatment with or without DDP (8 μg/ml) for 24 h, and the apoptosis level was measured through flow cytometry (E) and western blotting (F). DMSO: solvent control of 10058‐F4. (G) SGC7901 cells stably overexpressing LNC942 were transfected with c‐Myc siRNAs and treated with different concentrations of DDP for 24 h, after which the cell viability was measured using the MTS assay. (H) SGC7901 cells stably overexpressing LNC942 were pre‐treated with 10058‐F4 (6.25 μg/ml) for 24 h and co‐treated with different concentrations of DDP for 24 h, after which the cell viability was measured using the MTS assay. (I and J) SGC7901 cells stably overexpressing LNC942 were transfected with c‐Myc siRNAs for 48 h and treated with DDP (1 μg/ml) for 24 h, after which the apoptotic cells were analysed through flow cytometry (I) and western blotting (J). (K and L) SGC7901 cells stably overexpressing LNC942 were pre‐treated with 10058‐F4 (6.25 μg/ml) for 24 h and co‐treated with DDP (1 μg/ml) for 24 h. Apoptotic cells were analysed through flow cytometry (K) and western blotting (L). (M) The size and number of 3D‐cultured spheroids of LNC942 overexpressed or control SGC7901 with MSI2 or c‐Myc inhibition were measured by a 3D culture system. Spheroid size >40 cells per case was counted by ImageJ as one spheroid. (N and O) Pluripotent transcription factors were analysed in LNC942 (N) or MSI2 (O)‐depleted cells by quantitative real‐time PCR (qRT‐PCR). Data in (A‐C), (E), (G‐I), (K), (M‐O) panels are represented as mean ± SD of the three independent experiments; the *p* value was determined using a two‐tailed unpaired Student's *t* test. ns, *p* > .05; *p < .05; **p < .01; ***p < .001

As an essential oncogenic transcription factor, c‐Myc involves reprogramming, proliferation, apoptosis and chemoresistance of various cancer cells.^40^, ^41^ In this study, c‐Myc was significantly up‐regulated in chemoresistant GC cells (Figure 5B,C). The inhibition of c‐Myc by using c‐Myc siRNAs (Figure 7A) or small‐molecule c‐Myc inhibitor 10058‐F4 (Figure 7B) was found to impair chemoresistance (Figure 7A,B) and increase drug‐induced apoptosis (Figure 7C‐F, Figure S7A,B). Conversely, c‐Myc overexpression was found to have opposite effects on chemosensitive GC cells (Figure S7C‐F). Notably, the impact of LNC942 overexpression on chemoresistance was attenuated upon c‐Myc silencing in SGC7901 cells (Figure 7G‐L, Figure S7G). Taken together, these data demonstrated that LNC942 mediates chemoresistance in GC by promoting c‐Myc expression.

c‐Myc is also vital for regulating stemness, which is considered in charge of chemotherapy resistance.^42^ Indeed, the genes regulated by LNC942 or MSI2 were enriched in the stem cell differentiation pathway through GSEA (Figure S7H), indicating that LNC942 might promote chemoresistance by maintaining the stemness of GC cells. Interestingly, the number and size of spheres were found to be decreased in SGC7901 cells compared with those in SGC‐R cells (Figure S7I). Consistently, LNC942 depletion dramatically reduced the oncosphere formation of SCG‐R cells (Figure S7J). Nevertheless, LNC942 overexpression increased the size and number of spheroids derived from SGC7901 cells, which were destroyed by MSI2 or c‐Myc inhibition (Figure 7M). Besides, LNC942 or MSI2 depletion significantly reduced the expressions of pluripotent transcription factors, including Sox2, Nanog and Oct4, compared with those in control cells (Figure 7N,O). To conclude, LNC942 up‐regulated the c‐Myc level, thus inducing the stemness feature of GC cells to promote chemoresistance.

### 3.8 Inhibition of MSI2 reverses the effects of LNC942 on DDP resistance in GC

**FIGURE 8:**
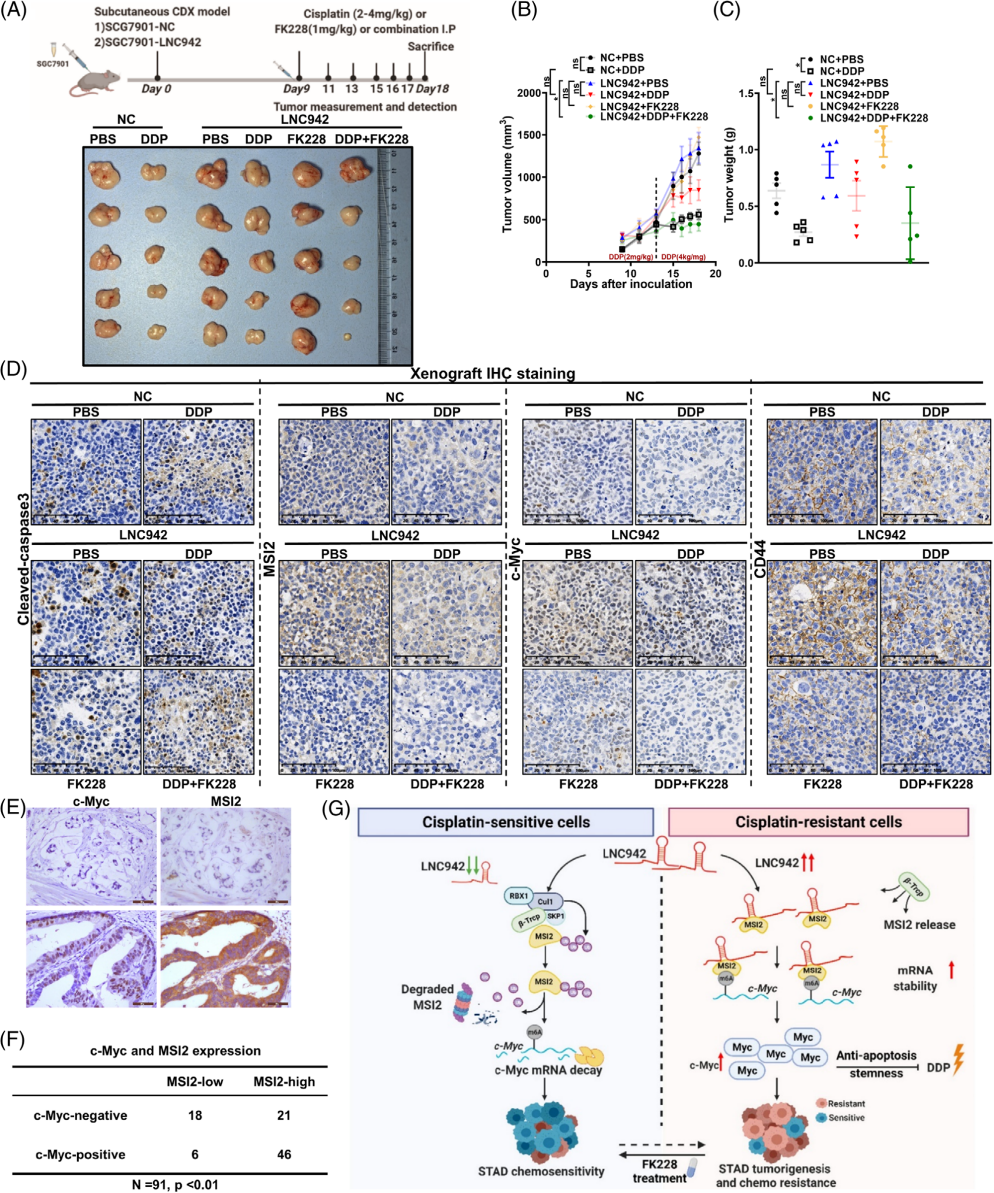
LNC942 promotes cisplatin (DDP) resistance by targeting MSI2 in vivo. (A) Xenografts in nude mice by inoculating SGC7901 cells that were stably overexpressing LNC942 or empty vector (NC) (*n* = 5/group). The administration of drug dose and time was recorded; representative photographs of tumours excised from the mice on day 18 are shown. (B) The growth curve showed changes in the tumour volume in mice from different groups; growth was assessed during the treatment period (days 9–18). (C) Tumour weight of the tumours excised from the mice in each group. (D) Representative immunohistochemical images of cleaved‐caspase 3, MSI2, c‐Myc and CD44 in excised tumour tissues. Scale bars = 100 μm. (E) The expression of MSI2 and c‐Myc in GC tissue was analysed by immunohistochemistry staining. (F) The correlation between MSI2 and c‐Myc expression in gastric cancer (GC) tissue was analysed by chi‐square test (*p* < .01). (G) Hypothetical model of LNC942 function in GC. High expression of LNC942 prevents the interaction between MSI2‐β‐Trcp and subsequently ubiquitin‐mediated degradation. Then, MSI2 stabilizes the *c*‐*Myc* mRNA stability in an m^6^A dependent manner. Increased expression of c‐Myc inhibits cell apoptosis and maintains stemness to promote DDP resistance. Data in B‐C are presented as the mean ± SEM; the *p* value was determined using a two‐tailed unpaired Student's *t* test. ns, *p* > .05; *p < .05; **p < .01; ***p < .001

To further support the in vitro findings and explore potential clinical applications, we established in vivo chemoresistant models. The nude mice injected with SGC7901 cells expressing LNC942 were significantly more resistant to DDP treatment than controls (NC). Moreover, combining FK228 with DDP strikingly decreased the tumour volumes of LNC942–SGC7901 cells, whereas the administration of DDP alone could not retard tumour growth. Notably, the tumour size and sensitization to DDP were comparable between the combination treatment group (DDP plus FK228) and the DDP‐treated NC group (Figure 8A‐C). Similar to in vitro results, immunohistochemical analysis results indicated an increased level of cleaved‐caspase 3 and a decreased level of MSI2 and c‐Myc in the combination‐treated group (Figure 8D), accompanied by a positive correlation between MSI2 and stemness markers, CD44 and c‐Myc (Figure 8D).^43^ Immunohistochemical analysis of GC tissue microarray showed that MSI2 level was positively correlated to c‐Myc level in GC patient tissues (Figure 8E,F). These findings suggested that the inhibition of LNC942/MSI2/c‐Myc could suppress tumour growth and be essential for regulating DDP chemotherapy efficacy even in chemoresistant GC cells in vivo.

## 4 DISCUSSION

Emerging evidence suggests the critical roles of lncRNAs in developing DDP resistance. For example, our previous study revealed that lncRNA ARHGAP5‐AS1 could facilitate chemoresistance in GC by activating the transcription and stabilizing the mRNA of its natural anti‐sense transcript ARHGAP5.^16^ Dysregulation of specific chemoresistant‐related lncRNAs has been discovered in human cancer, but the functional mechanisms of the majority lncRNAs in cancers remain unidentified. This study found that LNC942 is a novel chemoresistance‐related lncRNA up‐regulated in human GC cells and promotes DDP resistance in vitro and in vivo.

LncRNAs participate in various cancer‐related processes depending on their localization and specific interactions with DNA, RNA and proteins, thus modulating the chromatin function, altering cytoplasmic mRNAs stability and translation and interfering with signaling pathways.^7^ LNC942, a lncRNA predominantly located in the cytoplasm, was reported to bind directly to a methyltransferase, METTL4, and promote METTL14‐mediated RNA m^6^A methylation in breast cancer.^21^ To identify other essential proteins regulated by LNC942 in chemoresistance, we performed a human proteome microarray and found its specific and strongest interaction with an RBP, MSI2. As an oncogenic protein, MSI2 promotes malignant phenotypes in GC and is related to a poor prognosis in patients with GC.^44^ However, the mechanism of chemoresistance regulation by MSI2^10^, ^45^ and the relevance of post‐translational modification to the function of MSI2 remains poorly understood. Our study suggests that LNC942, which acted as a novel MSI2‐interacting partner, directly bound the RRM1 domain of MSI2 via its internal region (1162‐2244 nt), therefore protecting MSI2 from degradation through blocking its interaction with SCF^β‐TRCP^ E3 ubiquitin ligase. Our findings indicated that lncRNAs stabilized MSI2 protein and provided new insights into the functional relationship between lncRNAs and post‐translational modification of interacting proteins. However, since LNC942 and β‐Trcp bind to the different motif of MSI2, the underlying mechanisms of LNC942 in regulating MSI2‐β‐Trcp interaction are worth further investigations.

As a predominant transcription factor, c‐Myc is centrally involved in the reprogramming, proliferation and chemoresistance of various cancer cells.^40^ A previous study found that MSI2 sustained a mixed‐lineage leukemia self‐renewal program by binding to and keeping efficient translation of *c‐Myc* mRNA.^34^ Here, we noted that MSI2 directly interacts with *c‐Myc* mRNA and enhances its mRNA stability through LNC942. Consistent with the role of m^6^A in mediating the mRNA fate, we proved that MSI2 stabilizes *c‐Myc* mRNA in an m^6^A‐dependent manner. Importantly, we found that, instead of other reported m^6^A readers (such as IGF2BPs and YTHDF2), MSI2 may function as a potential m^6^A‐binding protein to recognize and stabilize *c‐Myc* mRNAs. As m^6^A preferably occurs on the DRACH motif,^46^ we searched the DRACH motif on the c‐Myc cDNA sequence, and 20 potential sites were found (not shown). However, additional sequence and structural features which determine the m^6^A modification sites have not been detected. Therefore, it is challenging to decide the exact m^6^A site for binding to MSI2. The m^6^A of *c*‐*Myc* mRNA was increased after knocking down LNC942 (Figure S6B). As MSI2 expression was decreased after silencing LNC942, it could be plausible that the *c*‐*Myc* m^6^A level was raised in a feedback manner. In all, further studies need to focus on clarifying the mechanisms for regulating *c‐Myc* mRNA m^6^A modifications.

To highlight the clinical relevance, we tried to inhibit MSI2 as a novel therapeutic avenue regarding the critical role of MSI2 in GC cancer development and chemoresistance. This study identified FK228, a peptide isostere of largazole,^47^ as a putative MSI2 inhibitor. Molecular docking and SPR assay verified the binding capacity between FK228 and MSI2. Besides, FK228 inhibits the MSI2 protein expression and the oncogenic RNA‐binding activity of MSI2 in GC cells. Although FK228 alone has a more negligible effect on inhibiting GC cell viability and tumour progression, combinations of FK228 with DDP chemotherapy have displayed synergistic effects both in vitro and in vivo. Therefore, this study provided the therapeutic potential of MSI2 inhibition for overcoming the acquired chemoresistance with LNC942/MSI2 overexpression. Indeed, more perspective analyses would be necessary to verify the clinical relevance of the LNC942‐MSI2‐c‐Myc axis in GC.

## 5 CONCLUSION

In summary, our results demonstrated that LNC942 plays a critical role in maintaining DPP resistance in GC. We noted that LNC942 functions on inhibiting β‐Trcp‐mediated MSI2 degradation interaction, which affects the expression of downstream gene targets of MSI2, such as *c‐Myc* mRNA. Furthermore, we identified MSI2 as a potential m^6^A‐recognized protein that could aid *c‐Myc* mRNA stability. Our finding indicating FK228 as a new MSI2 inhibitor can provide a strategy to overcome DDP resistance. Cumulatively, our findings suggest the potential of targeting the LNC942‐MSI2‐c‐Myc axis in overcoming chemoresistance in patients with GC.

## CONFLICT OF INTEREST

The authors declare that they have no conflict of interest.

## Supporting information

Supporting Information
