# Erratum for the Clinical and Translational Medicine ‘LncRNA LINC00942 promotes chemoresistance in gastric cancer by suppressing MSI2 degradation to enhance c‐Myc mRNA stability’ by Yiran Zhu et al.

**DOI:** 10.1002/ctm2.70106

**Published:** 2024-11-22

**Authors:** Yiran Zhu, Bingluo Zhou, Xinyang Hu, Shilong Ying, Qiyin Zhou, Wenxia Xu, Lifeng Feng, Tianlun Hou, Xian Wang, Liyuan Zhu, Hongchuan Jin

**Affiliations:** ^1^ Laboratory of Cancer Biology Key Lab of Biotherapy in Zhejiang Province, Cancer Center of Zhejiang University Sir Run Run Shaw Hospital School of Medicine, Zhejiang University Hangzhou Zhejiang China; ^2^ Department of Medical Oncology, Sir Run Run Shaw Hospital, School of Medicine Zhejiang University Hangzhou Zhejiang China; ^3^ Department of Clinical Medicine Wenzhou Medical University Wenzhou Zhejiang China

Following the publication of the original article,^1^ the authors identified a minor error in Figure 1G, where the western blot image of the a‐tubulin reference for SGC‐R was inadvertently duplicated with that of BGC‐R. We sincerely apologise for any confusion this may have caused. It is important to note that this error did not affect the overall results, as the cleavage of PARP1 and caspase‐3 was appropriately supported by the levels of total PARP1 and caspase‐3.

We have located the original western blot data for the a‐tubulin reference for SGC‐R and have made the necessary corrections to Figure 1G. Importantly, we assure readers that this erratum does not impact the conclusions or descriptions presented in the article.

1. Zhu, Y., Zhou, B., Hu, X., Ying, S., Zhou, Q., Xu, W., Feng, L., Hou, T., Wang, X., Zhu, L., & Jin, H. (2022, Jan). LncRNA LINC00942 promotes chemoresistance in gastric cancer by suppressing MSI2 degradation to enhance c‐Myc mRNA stability. *Clin Transl Med, 12*(1), e703. https://doi.org/10.1002/ctm2.703

**FIGURE 1:**
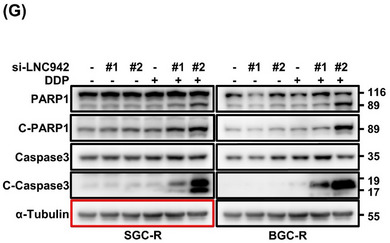
Original Figure 1 (G) with red block:Apoptotic cells among LNC942 knocked‐down SGC‐R and BGC‐R cells were treated with DDP (8 µg/ml) for 24 h and then analysed by anti‐cleaved‐PARP1 and cleaved‐caspase 3 through western blotting.

**FIGURE 1:**
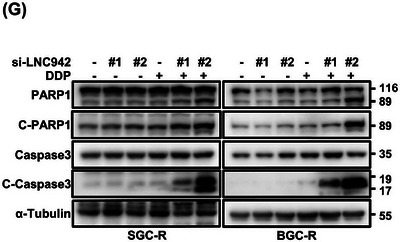
Updated Figure 1 (G): Apoptotic cells among LNC942 knocked‐down SGC‐R and BGC‐R cells were treated with DDP (8 µg/ml) for 24 h and then analysed by anti‐cleaved‐PARP1 and cleaved‐caspase 3 through western blotting.

