# Supplementary material for: LncRNA LINC00942 promotes chemoresistance in gastric cancer by suppressing MSI2 degradation to enhance c‐Myc mRNA stability
Source: Clin Transl Med. 2022 Jan 24;12(1):e703. doi: 10.1002/ctm2.703 (PMC8785984; doi:10.1002/ctm2.703)
Supplement: Supplementary file 1 — Supporting Information [file CTM2-12-e703-s001.docx]

**LncRNA LINC00942 promotes chemoresistance in gastric cancer by suppressing MSI2 degradation to enhance *c-Myc* mRNA stability**

Yiran Zhu^1^, Bingluo Zhou^1^, Xinyang Hu^1^, Shilong Ying^1^, Qiyin Zhou^2^, Wenxia Xu^1^, Lifeng Feng^1^, Tianlun Hou^3^, Xian Wang^2^, Liyuan Zhu^1,*^, Hongchuan Jin^1,*^

^1^Laboratory of Cancer Biology, Key Lab of Biotherapy in Zhejiang Province, Cancer Center of Zhejiang University, Sir Run Run Shaw Hospital, School of Medicine, Zhejiang University, Hangzhou, Zhejiang, China; ^2^Department of Medical Oncology, Sir Run Run Shaw Hospital, School of Medicine, Zhejiang University, Hangzhou, Zhejiang, China; ^3^Department of Clinical Medicine, Wenzhou Medical University, Wenzhou, Zhejiang, China.

***Correspondence to:** Dr. Liyuan Zhu, email:0016644@zju.edu.cn and Dr. Hongchuan Jin, email: jinhc@zju.edu.cn.

Supplemental Figures and Figure legends


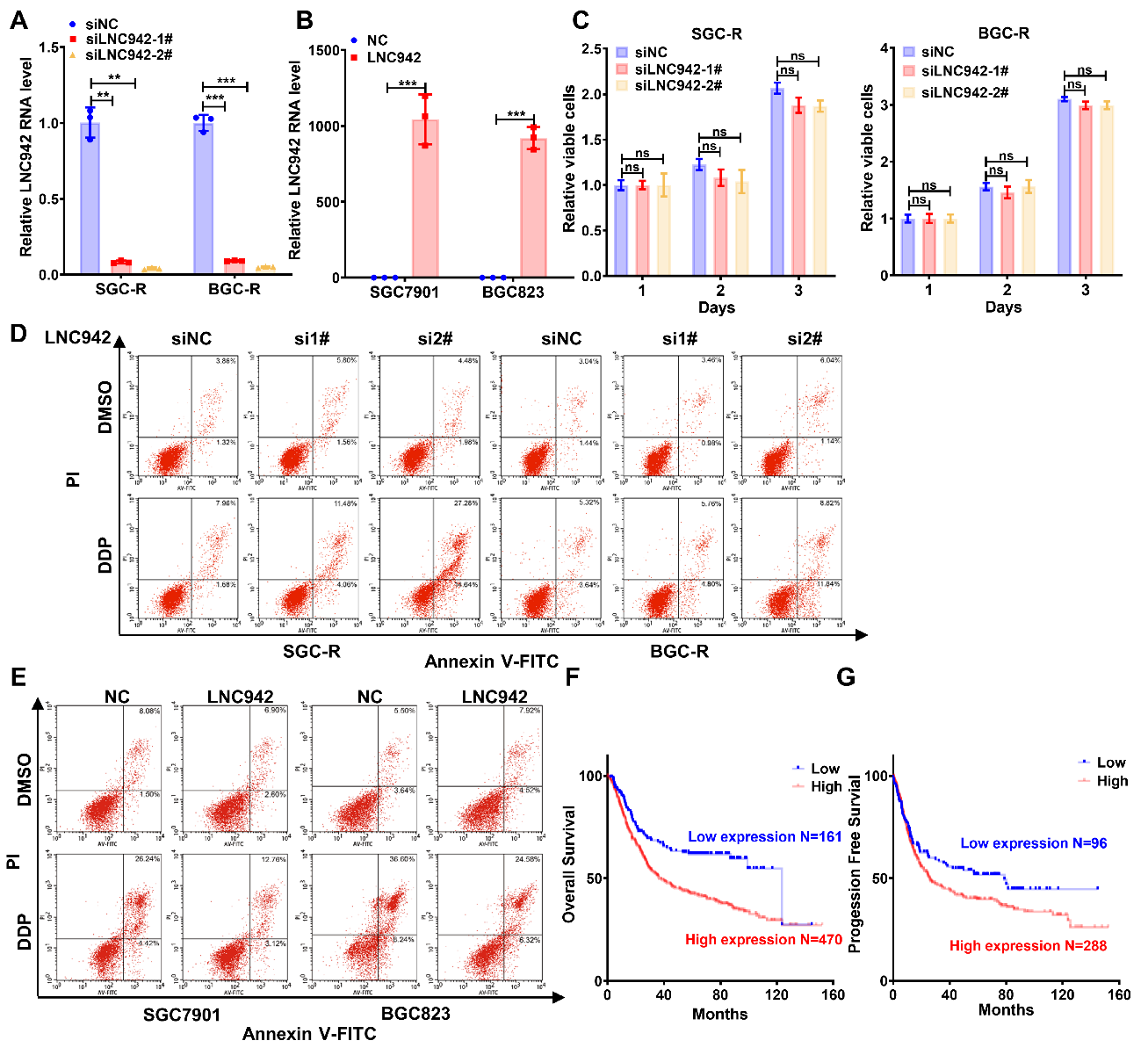


Figure S1.LNC942 promotes gastric cancer cell viability and inhibits cell apoptosis.

(A-B)The efficiency of knock-down of LNC942 by two siRNAs in SGC-R and BGC-R (A), or cells stable overexpression of LNC942 in SGC7901 and BGC823 cells, was verified by qRT-PCR (B). (C) Total viable cells were measured by MTS assay on the indicated days upon LNC942 knock-down in chemoresistant cells. Data were presented relative to day 1. (D)The raw flow cytometry apoptosis signal of resistant cells after transfecting si-LNC942 #1 or #2 or si-NC and DDP (8 μg/mL) treatment for 24 h. (E) The raw flow cytometry apoptosis signal of LNC942 stable-overexpressed sensitive cells with DDP (1μg/mL) treatment for 24 h. NC, empty vector. (F) Overall survival in TCGA cohort according to LNC942 expression was analyzed by Kaplan–Meier analysis (n = 631, log-rank test, two-sided). (G) Progression‑free survival analysis based on LNC942 levels in STAD (TCGA, n = 339, log-rank test, two-sided). Data in A-C are determined by a two-tailed unpaired Student’s *t-test*. ns, p > 0.05; *p < 0.05; **p < 0.01; ***p < 0.001.


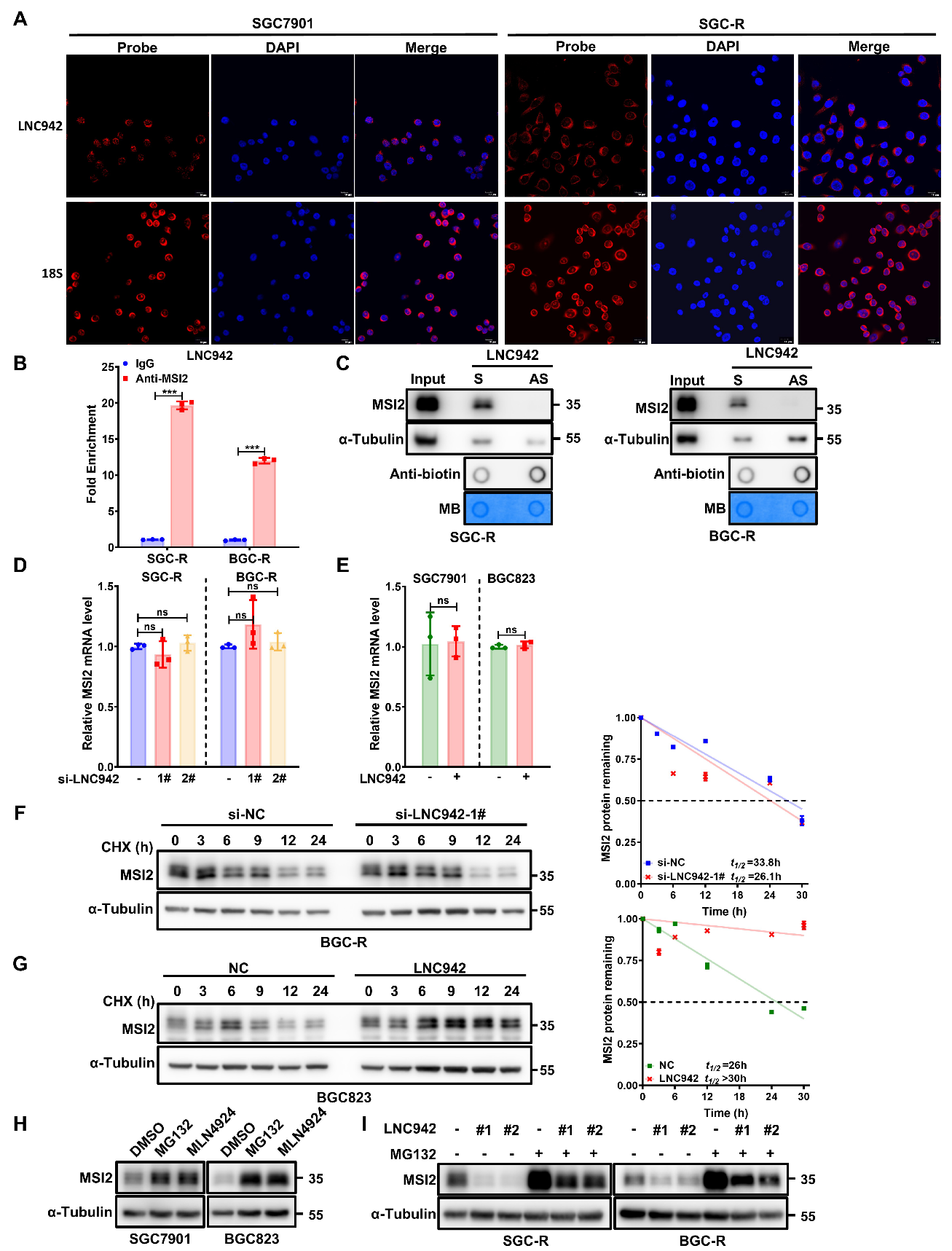


Figure S2. LNC942 affects MSI2 stability by preventing its ubiquitination.

(A) RNA FISH assays confirmed that LNC942 was mainly distributed in the cytoplasm of SGC7901 (left) and SGC-R (right) cells. Blue: DAPI; Red: Cy3-labeled LNC942 or 18S probes. 18S: cytoplasmic control. All scale bars: 10 μm. (B) RIP assays indicated that LNC942 precipitated with MSI2. Fold enrichment represented the RNA levels associated with MSI2 relative to the IgG (negative control). (C) MSI2 in cell lysates was pulled-down by biotin-labeled LNC942 but not by LNC942 antisense RNA. S, sense. AS, antisense. LNC942 probes were quantified (300 ng) and detected by the dot blot assay. MB: methylene blue, used as the internal reference. (D) qRT-PCR analysis of the mRNA level of MSI2 in SGC-R or BGC-R cells transfected with si-NC or LNC942 siRNAs. (E) qRT-PCR detection of MSI2 expression in SGC7901 or BGC823 cells stably expressing LNC942. (F) LNC942 knock-down shortened the half-life of MSI2 in BGC-R cells, as indicated by Western blotting. MSI2 protein abundance was quantified by Image J. (G)Western blotting analysis of MSI2 protein half-life in LNC942 stably expressed BGC823 cells with CHX (100 µg/mL) for the indicated time points. MSI2 protein abundance was quantified by ImageJ. (H) Western blotting analysis of the MSI2 level in SGC7901 and BGC823 cells treated with MG-132 (20 μM) or MLN4924 (1 μM) for 24 h before harvesting. (I) MSI2 downregulation by LNC942 knock-down was abolished by MG-132 (20μM, 24 h). Data in B, D-G are shown as mean ± SD of three independent experiments. The *P* value was determined using a two-tailed unpaired Student’s *t -test*. ns, p > 0.05; *p < 0.05; **p < 0.01; ***p < 0.001.


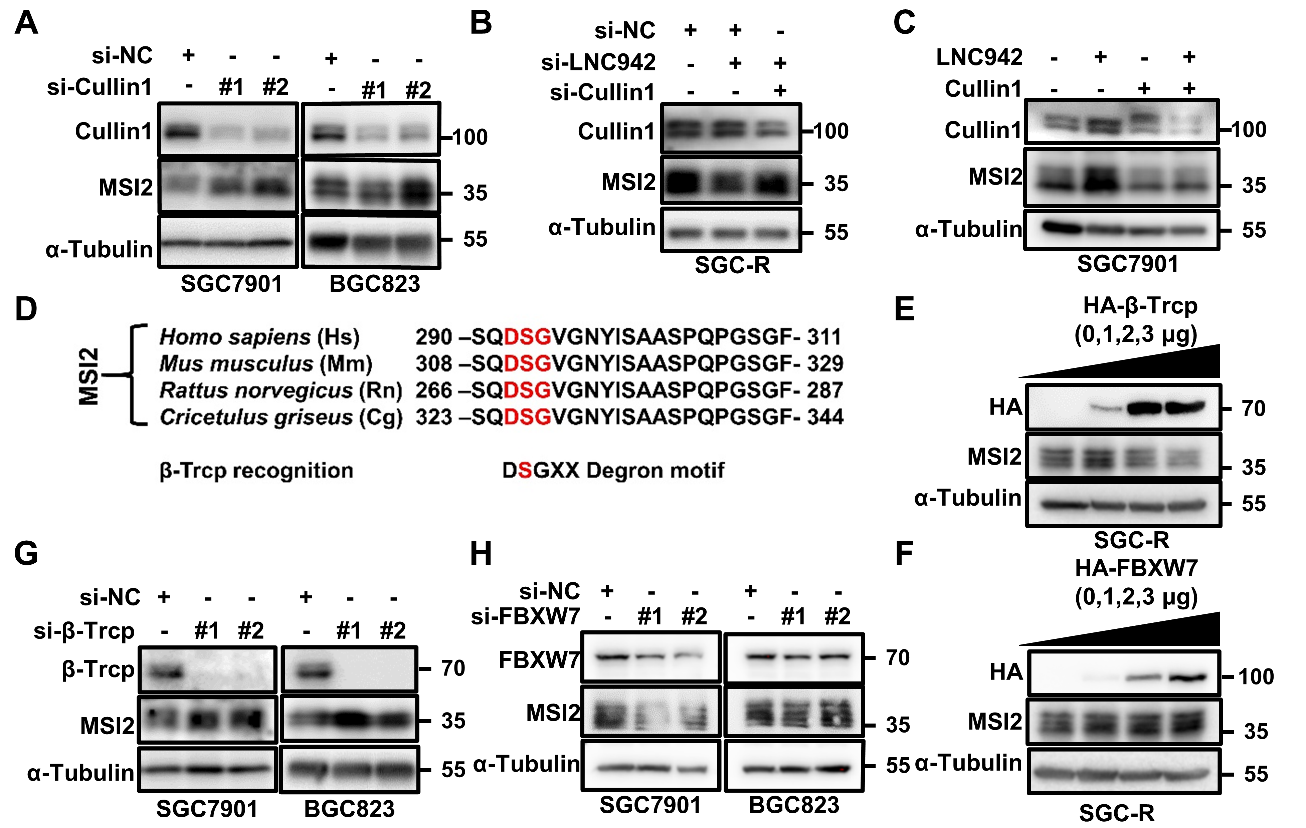


Figure S3. LNC942 prevents SCF^β-TRCP^ E3 ubiquitin ligase-mediated MSI2 ubiquitination.

**(A)** Western blotting detection of MSI2 levels in SGC7901 and BGC823 cells after knock-down Cullin1. **(B)** SGC-R cells were co-transfected with LNC942 and Cullin1 siRNAs, followed by Western blot analysis of MSI2 and Cullin1 expression. **(C)** SGC7901cells were co-transfected with LNC942 and Cullin1 plasmids, followed by Western blot analysis of MSI2 and Cullin1 expression. **(D)** Alignment of the candidate degron sequence in MSI2 for β-TrCP binding. **(E-F)** Western blotting analysis of MSI2 protein level in SGC-R cells transfected with HA-β-Trcp (E) or HA-FBXW7 (F) plasmids as indicated. **(G-H)**Western blotting detection of MSI2 levels in SGC7901 and BGC823 cells after knock-down β-Trcp (G) or FBXW7 (H).


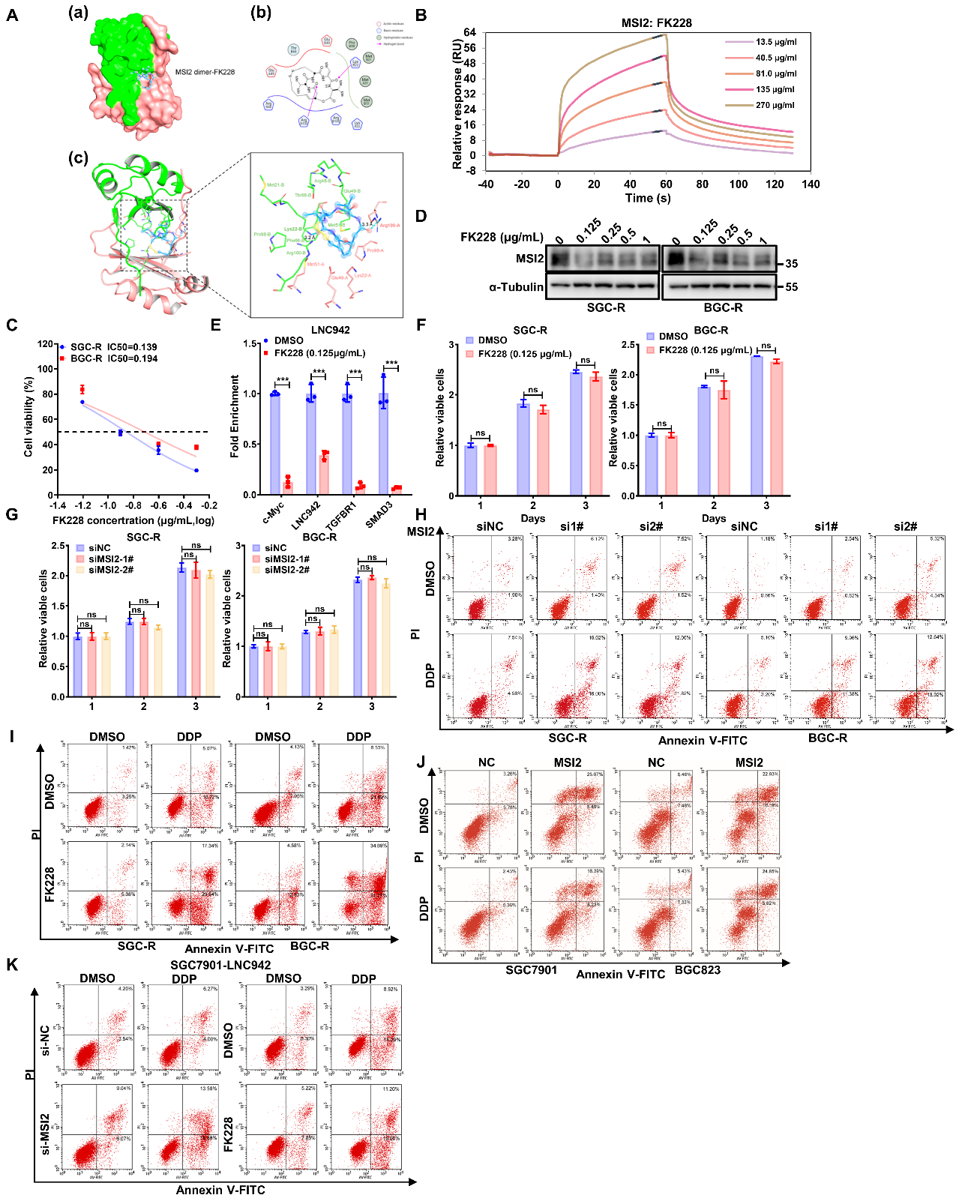


Figure S4. MSI2 promotes gastric cancer cell viability and inhibits cell apoptosis.

**(A)** The predicted binding mode between FK228 and MSI2. (a) FK228 in the binding site of MSI2 is represented as surface. (b) 2D interaction of FK228 in the binding site of MSI2. (c) 3D interaction of FK-228 in the binding site of MSI2. **(B)** SPR analysis of FK228 binding to immobilized full-length MSI2 protein. Five dosages were used. **(C)** The IC_50_ of FK228 (24h) was measured by MTS assay. **(D)**The efficiency of FK228 on MSI2 expression was tested by Western blotting in SGC-R and BGC-R cells. **(E)** FK228 inhibitory effect on the RIP enrichment of MSI2 mRNA targets in SGC-R cells after 24 h treatment at 0.125μg/mL. **(F)** Total viable cells treated with FK228 (0.125μg/mL, 24 h) were measured by MTS assay on the indicated days in chemoresistant cells. Data were presented relative to day 1. **(G)** The MTS assay measured total viable cells on the indicated days after transfecting MSI2 siRNAs (24 h) in chemoresistant cells. **(H)** The raw flow cytometry apoptosis signal of resistant cells after transfecting siMSI2 #1 or #2 or siNC for 48 h and DDP (8μg/mL) treatment for 24 h. **(I)** The raw flow cytometry apoptosis signal of resistant cells with DDP (8μg/mL), FK228 (0.125μg/mL) or co-treat for 24 h. **(J)** The raw flow cytometry apoptosis signal of MSI2 overexpressed sensitive cells with DDP (1μg/mL) treatment for 24 h. **(K)** The raw flow cytometry apoptosis signal of LNC942 stably expressed SGC7901 cells transfected with MSI2 siRNAs (left panel) that treated with DDP (1μg/mL) treatment for 24 h, or co-treated with FK228 (0.125μg/mL) and DDP (1μg/mL) for 24 h (right panel). Data in **C, E-G** are shown as mean ± SD of three independent experiments. The *P* value was determined by a two-tailed unpaired Student’s *t-test*. ns, p > 0.05; *p < 0.05; **p < 0.01; ***p < 0.001.


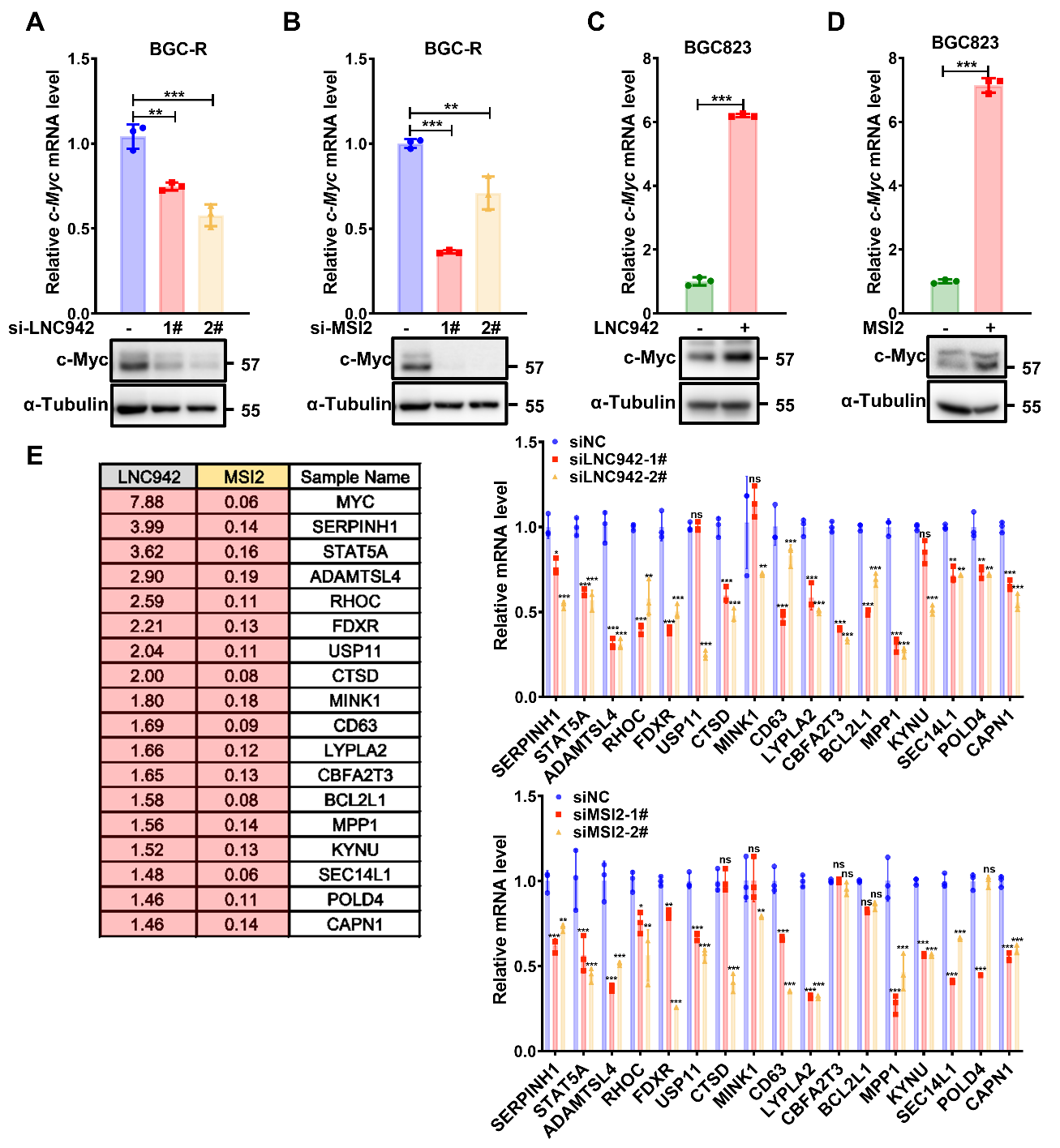


Figure S5. LNC942 increases MSI2 downstream c-Myc expression.

**(A, B)** LNC942 (A) or MSI2 (C) siRNAs downregulated c-Myc expression in BGC-R cells, as determined using qRT-PCR (top panel) and Western blotting (bottom panel). **(C)** Stable expression of LNC942 increases the c-Myc level in BGC823 cells, determined by qRT-PCR (top panel) and Western blotting (bottom panel). **(D)** Ectopic expression of MSI2 increases the c-Myc level in BGC823 cells, determined by qRT-PCR (top panel) and Western blotting (bottom panel). **(E)** RNA expression of the c-Myc target genes. The c-Myc target genes downregulated after LNC942 and MSI2 knock-down were enriched by GSEA and listed according to the rank metric score. The mRNA expression of them was determined by qRT-PCR. Data in **A-E** are shown as mean ± SD of three independent experiments. The *P* value was determined by a two-tailed unpaired Student’s *t-test*. ns, p > 0.05; *p < 0.05; **p < 0.01; ***p < 0.001.


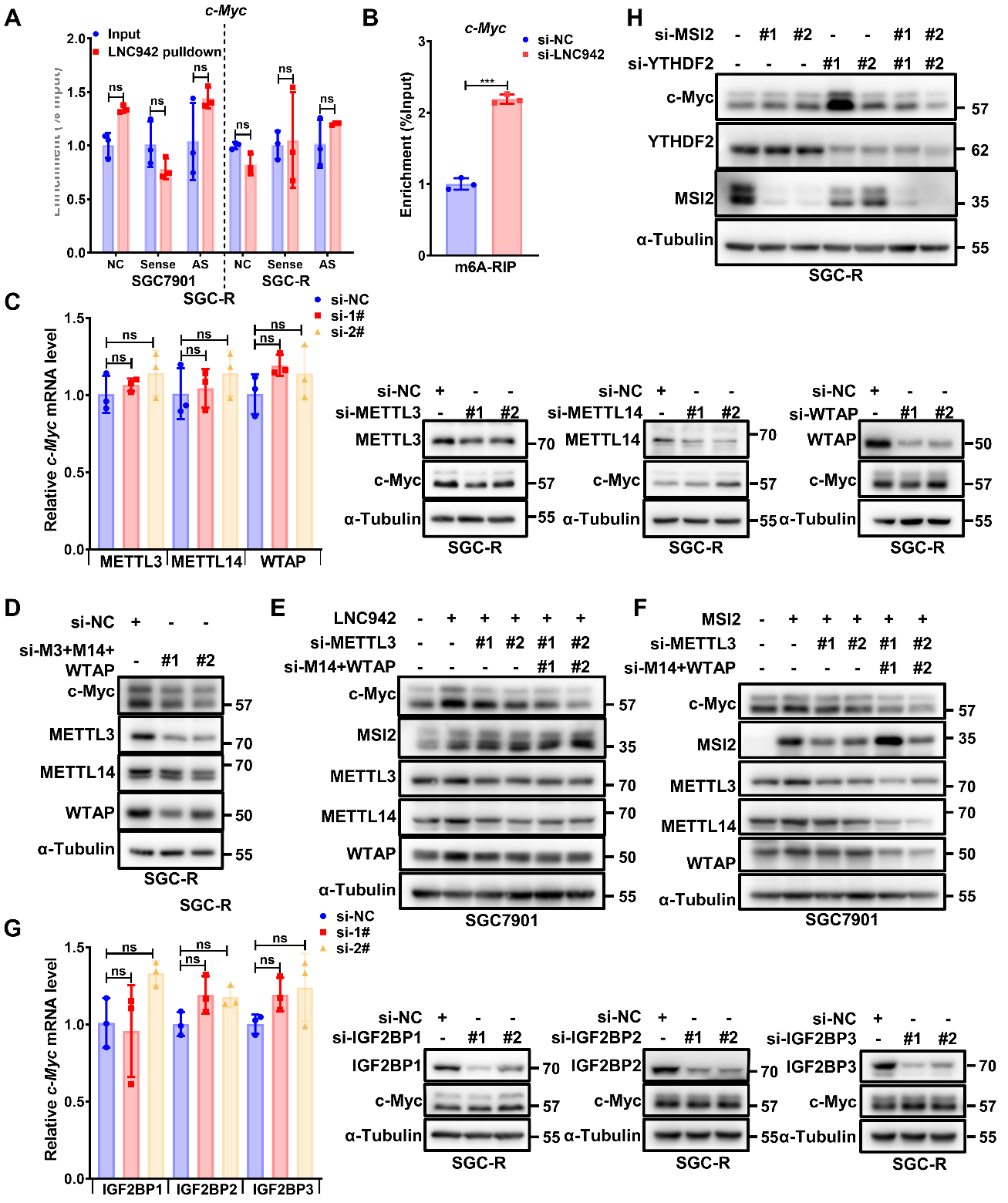


Figure S6. The effect of changing m^6^A-related regulators on c-Myc expression.

**(A)** SGC7901 and SGC-R cell lysates were incubated with biotin-labeled sense LNC942 probe (LNC942 pulldown), GFP-labeled sense probe (NC, negative pulldown) and antisense probe (AS, control pulldown); after pulldown, RNA was extracted, and *c-Myc* mRNAs were assessed by RT-qPCR. **(B)** RIP-qRT-PCR analysis of the m^6^A modifications of *c-Myc* in LNC942 knock-down or control SGC-R cells. **(C)** The qRT-PCR and Western blotting detection of the mRNA and protein level of c-Myc in SGC-R cell with or without METTL3, METTL14 or WTAP knock-down. **(D)** The protein level of c-Myc in SGC-R cell with or without METTL3-METTL14-WTAP complex knock-down was detected using Western blotting. **(E-F)** LNC942 (E) or MSI2 (F) overexpressed SGC7901 cells were transfected with METTL3 or METTL3+METTL14+WTAP siRNAs, the protein level of c-Myc was measured using Western blotting. **(G)** The qRT-PCR and Western blotting detection of the mRNA and protein level of c-Myc in SGC-R cell upon IGF2BP1or IGF2BP2 or IGF2BP3 silencing. **(H)** Changes of c-Myc protein level in MSI2 or/and YTHDF2 knock-down SGC-R cells. Data in **A-C** and **G** are shown as mean ± SD of three independent experiments. The *P* value was determined by a two-tailed unpaired Student’s *t-test*. ns, p > 0.05; *p < 0.05; **p < 0.01; ***p < 0.001.


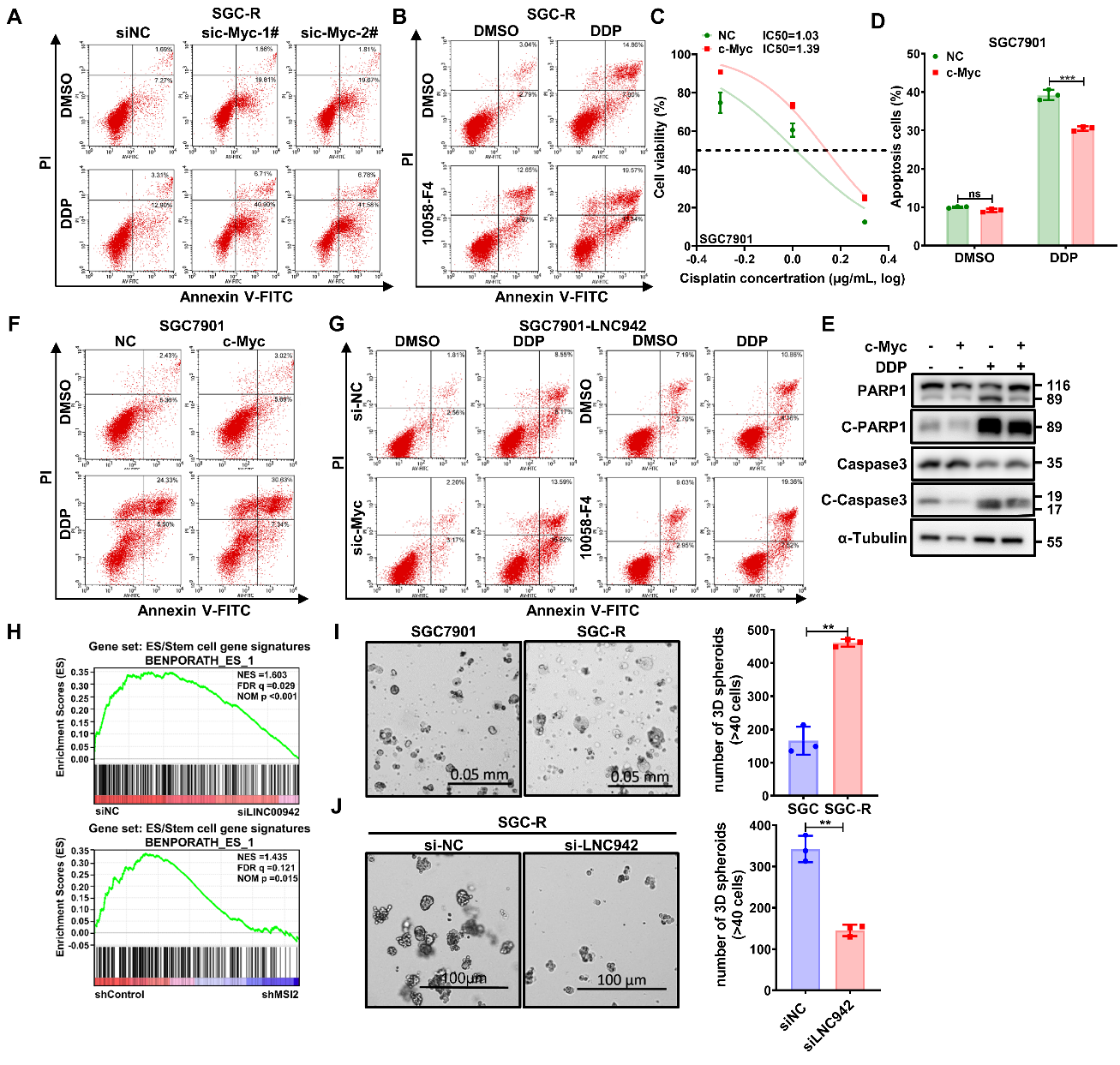


Figure S7. c-Myc inhibits GC cell apoptosis and stemness.

**(A)** The raw flow cytometry apoptosis signal of SGC-R cells after transfecting sic-Myc #1 or #2 or si-NC and DDP (8μg/mL) treatment for 24 h. **(B)** The raw flow cytometry apoptosis signal of SGC-R cells pretreatment with 10058-F4 (6.25μg/mL) for 24 h followed by treatment with or without DDP (8μg/mL) for 24 h. **(C)**The c-Myc plasmid or empty vector (NC) were transfected into SGC7901 cells, and relative cell viability before or after DDP(1μg/mL) treatment for 24 h was detected using MTS assay. **(D-E)** The apoptosis of sensitive cells with or without c-Myc overexpression in the presence or absence of DDP treatment (1μg/mL, 24 h) was measured using flow cytometry (D) as well as Western blotting (E). **(F)**The raw flow cytometry apoptosis signal of c-Myc overexpressed sensitive cells treatment with DDP (8μg/mL) for 24 h. **(G)**The raw flow cytometry apoptosis signal of stable LNC942 overexpressed SGC7901 cells after silencing c-Myc using siRNAs or 10058-F4 (6.25μg/mL) and DDP (1μg/mL) treatment for 24 h. **(H)** GSEA of ES/Stem cell gene signature in the expression profiles of SGC-R cells expressing LNC942 siRNAs or shMSI2 expression profile expression profiles from the GEO database (GSE69583). **(I)** The size and number of 3D-cultured spheroids of SGC7901 and SGC-R cells were measured by a 3D culture system. **(J)** 3D-cultured mammosphere model was used to measure the effect of LNC942 knock-down on the stemness of SGC-R cells. The number of spheroids was calculated by Image J. Data in **C, D, I** and **J** are shown as mean ± SD of three independent experiments. The *P* value was determined by a two-tailed unpaired Student’s *t-test*. ns, p > 0.05; *p < 0.05; **p < 0.01; ***p < 0.001.

**Supplementary Tables**

Table S1. SiRNA sequences

| **siRNA** | **Sequences(5'-3')** |
| --- | --- |
| LNC942-1# | GUCUGCGGGAAACAGUACUTT |
| LNC942-2# | GGAGCUAAUCCAGAAGGAATT |
| MSI2-1# | CUCACCAGAUAGCCUUAGATT |
| MSI2-2# | GACCCAGCAAGUGUAGAUATT |
| c-Myc-1# | CACCUAUGAACUUGUUUCATT |
| c-Myc-2# | GCCGUAUUUCUACUGCGACTT |
| Cullin1-1# | GCUCUACACUCAUGUUUAUTT |
| Cullin1-2# | GAACCCAGUUACUGAAUAUTT |
| β-Trcp-1# | GUGGAAUUUGUGGAACAUCTT |
| β-Trcp-2# | GUGGAAUUUGUGGAACAUCdTdT |
| FBXW7-1# | GCACACUGCAAGGAAUGGUTT |
| FBXW7-2# | GGAGUAUGGUCAUCACAAATT |
| METTL3-1# | GCAAGAAUUCUGUGACUAUTT |
| METTL3-2# | GCUGCACUUCAGACGAAUUTT |
| METTL14-1# | GGCUAAAGGAUGAGUUAAUTT |
| METTL14-2# | GGACUUGGGAUGAUAUUAUTT |
| WTAP-1# | CACAGAUCUUAACUCUAAUTT |
| WTAP-2# | GGGAAAACAUCCUUGUAAUTT |
| IGF2BP1-1# | AGUGGUGAAUGUCACCUAUTT |
| IGF2BP1-2# | UGGCCCAUAAUAACUUUGUTT |
| IGF2BP2-1# | AUAUACAACCCGGAAAGAATT |
| IGF2BP2-2# | GAUCUUUGGGAAACUGAAATT |
| IGF2BP3-1# | AGGAAUUGACGCUGUAUAATT |
| IGF2BP3-2# | CUCAUUCUUAUUUCAAGAUTT |
| YTHDF2-1# | CCUACCAGAUGCAAUGUUUTT |
| YTHDF2-2# | GCUCCUGGCAUGAAUACUATT |

Table S2. Primers of recombinant DNA

| **Primers** | **Sequences(5'-3')** |
| --- | --- |
| Flag/MSI2-FL,T1,2-F | GCTCTAGAATGGAGGCAAATGGGAGC |
| Flag/MSI2-T1-R | CGGGATCCTTTCGGCTGAGCTTTCTTAC |
| Flag/MSI2-T2-R | CGGGATCCTATTTTCTTTGTTCTTGTGACCATC |
| Flag/MSI2-T3-F | GCTCTAGAAAAGAAGTCATGTTCCCACCTG |
| Flag/MSI2-T4-F | GCTCTAGAAAGAAAATATTTGTAGGCGGGTTATC |
| Flag/MSI2-T5-F | GCTCTAGACCCGGTAAAATGTTTATCGGTG |
| Flag/MSI2-FL,T3,4,5-R | CGGGATCCTCAATGGTATCCATTTGTAAAGGC |
| Myc-MSI2-F | GGGCTGCAGGAATTCGATATCATGGAGACTGACGCGCCC |
| Myc-MSI2-R | GTCGACGGTATCGATAAGCTTTCAGTGGTACCCATTGGTGAAGG |
| Flag/c-Myc-F | GCTCTAGAATGCCCCTCAACGTTAGCTTC |
| Flag/c-Myc-R | CGGGATCCCGCACAAGAGTTCCGTAGCTG |

Table S3. Primer sequences used in qRT-PCR assays

| **Target gene** | **Primer sequences(5'-3')** | |
| --- | --- | --- |
|  | **Forward** | **Reverse** |
| LNC942 | GGTGTCTGCGGGAAACAGTAC | GAACAAAGAGTCAGGTTGTGTGG |
| MSI2 | GCTCTAGAATGGAGGCAAATGGGAGC | CGGGATCCTCAATGGTATCCATTTGTAAAGGC |
| c-Myc | GCCTCAGAGTGCATCGAC | TCCACAGAAACAACATCG |
| β-actin | ACTCTTCCAGCCTTCCTTCC | CGTCATACTCCTGCTTGCTG |
| Malat1 | CTTCCCTAGGGGATTTCAGG | GCCCACAGGAACAAGTCCTA |
| GAPDH | AAGGTCGGAGTCAACGGATTTG | CCATGGGTGGAATCATATTGGAA |
| BCAT1 | TGGAGAATGGTCCTAAGCTG | GCACAATTGTCCAGTCGCTC |
| 18S rRNA | GTAACCCGTTGAACCCCATT | CCATCCAATCGGTAGTAGCG |
| SERPINH1 | TCAGTGAGCTTCGCTGATGAC | CATGGCGTTGACTAGCAGGG |
| STAT5A | CGACGGGACCTTCTTGTTG | GTTCCGGGGAGTCAAACTTCC |
| ADAMTSL4 | GAGAGTCTGTCGGCTGAAGG | CCCCAGGGGTGTCTGATAAAA |
| RHOC | CCTGAGGCAAGACGAGCAC | GATCCGGTTCGCCATGTCC |
| FDXR | TTCTCCACACAGGAGAAGACC | CAAGGCTTCCACCCTCTTTAG |
| USP11 | CAGCTGCTTGGCATTACCTG | GCAGCTCTATGACCTTGCGT |
| CTSD | ATTCAGGGCGAGTACATGATCC | CGACACCTTGAGCGTGTAG |
| MINK1 | GCTGAGGTCAAGCTAGTGGAT | CCAATGAAAGTGTTCCGTCTGC |
| CD63 | GTTGGCACGGAGGCCC | GCAGGAGGACGTAGAGCAAG |
| LYPLA2 | CCAGGGAGTGGAGGCCC | GTGAGCAGGGGCACAGAC |
| CBFA2T3 | CACTCACCAACAGCCATCAAT | CGTCAATGTCGAGTTCACCAG |
| BCL2L1 | CCTAAGGCGGATTTGAATCTCTTTC | CTCACTGAGTCTCGTCTCTGGT |
| MPP1 | AGGACATGTACACCAACGGG | TTCAGCGTGATTCCCTCCTG |
| KYNU | CGATTAAACCTGCGAGATCGG | GTCTCTCTAAAGCTCTTGTCCTTG |
| SEC14L1 | GTGGAACCGAGACTGCCC | AGCCCGAAGTCTAGCTCTTG |
| POLD4 | GCACCGTCTCTGGCATCTC | GAGGTTCTTGGGTGGTGAGC |
| CAPN1 | CGGCCCCTCCTCAGAGC | TTCTGCACTTGGGCTGACAC |

Table S4. Differentially expressed LncRNAs in resistant cells vs. sensitive cells (Top100)

| **Normalized Intensity of each sample (log2 transformed)** | | | | |
| --- | --- | --- | --- | --- |
| **GeneSymbol** | **SGC-R** | **SGC7901** | **BGC-R** | **BGC823** |
| ***LINC00942*** | ***9.435102*** | ***6.8759227*** | ***12.18571*** | ***6.555476*** |
| AK025793 | 7.76475 | 4.5473375 | 9.487736 | 5.0050936 |
| BX571672.2 | 11.887837 | 8.014138 | 11.0293665 | 6.6694145 |
| LOC375196 | 8.336904 | 6.516346 | 9.779133 | 5.4722815 |
| BX004987.5 | 12.208439 | 8.323728 | 11.379971 | 7.083057 |
| TIPARP-AS1 | 6.8743157 | 3.967215 | 8.703135 | 5.3113394 |
| XLOC_008233 | 8.825081 | 6.3845286 | 9.786268 | 6.709053 |
| LINC00478 | 7.804808 | 6.079855 | 7.6802154 | 4.6904287 |
| AC002454.1 | 7.660896 | 4.963239 | 6.2689686 | 3.2799034 |
| XLOC_012542 | 9.969334 | 6.4474564 | 7.911144 | 4.9425797 |
| XXbac-BPG55C20.7 | 6.6257696 | 4.5167894 | 7.406636 | 4.582178 |
| VTRNA1-2 | 8.39884 | 5.8248186 | 11.146394 | 8.368599 |
| ARHGAP5-AS1 | 7.5625362 | 5.225907 | 9.192132 | 6.4433937 |
| AC121336.2 | 6.162752 | 4.1027 | 8.201796 | 5.478926 |
| RP11-150O12.3 | 7.3545713 | 3.6111832 | 8.375351 | 5.6600714 |
| RP11-318A15.2 | 6.4861555 | 4.613723 | 7.3989143 | 4.780348 |
| FLJ41278 | 7.3209853 | 5.310731 | 6.7153096 | 4.104513 |
| GSTM3 | 10.9057455 | 8.650833 | 11.6622715 | 9.086645 |
| ANKRD20A11P | 9.997922 | 7.4235773 | 9.578276 | 7.012877 |
| LOC84989 | 10.040408 | 7.283554 | 9.339682 | 6.784381 |
| BX248273 | 6.4712973 | 4.396601 | 7.108347 | 4.622893 |
| RP11-504G3.1 | 10.053556 | 8.092654 | 11.393158 | 9.042363 |
| RP11-429G19.2 | 5.502203 | 3.3302023 | 8.024252 | 5.7062654 |
| RP11-818F20.5 | 9.889731 | 8.036238 | 12.183756 | 9.908104 |
| RP11-53O19.1 | 6.5283904 | 4.7048674 | 7.8592486 | 5.618522 |
| LINC00472 | 7.970665 | 4.418353 | 8.2549715 | 6.021758 |
| DLEU2 | 6.0796375 | 4.218947 | 6.995473 | 4.830093 |
| RP11-213H15.3 | 6.16501 | 3.54689 | 8.213257 | 6.0689697 |
| AC067945.2 | 5.019492 | 2.345003 | 7.3488793 | 5.309875 |
| RP11-752G15.9 | 5.496585 | 2.9967914 | 6.6014295 | 4.629319 |
| AK298056 | 6.1219683 | 3.839687 | 6.1664534 | 4.5167065 |
| RP11-15E18.1 | 7.381425 | 5.2168517 | 7.9039865 | 6.2840557 |
| RP4-660H19.1 | 6.7285433 | 4.503335 | 6.7795277 | 5.1802454 |
| ANKRD36B | 9.060002 | 6.909711 | 9.500191 | 7.9590654 |
| AC140481.7 | 6.199713 | 3.9486022 | 5.894316 | 4.3775597 |
| CYP4F43P | 8.441345 | 5.3369246 | 7.924052 | 6.407964 |
| FHL1 | 6.801156 | 4.532896 | 6.2034726 | 4.7413955 |
| XLOC_001643 | 6.5113516 | 3.625578 | 7.489098 | 6.0450163 |
| XLOC_002133 | 7.754633 | 4.802282 | 6.474765 | 5.162234 |
| BX571672.5 | 6.626082 | 2.345003 | 7.000262 | 5.787819 |
| RP11-1038A11.1 | 6.909109 | 4.66155 | 5.648798 | 4.449482 |
| LOC729421 | 4.9602532 | 2.871435 | 5.6336308 | 4.4588566 |
| RP1-80B9.2 | 6.0252247 | 2.8986044 | 5.5734243 | 4.4004097 |
| RP11-22A3.2 | 6.9388742 | 4.846608 | 6.9200196 | 5.762991 |
| C17orf76-AS1 | 13.003122 | 10.074449 | 10.182535 | 9.03997 |
| LPP-AS2 | 6.617207 | 4.3391414 | 7.2933216 | 6.1749487 |
| LOC100130950 | 6.400807 | 4.292069 | 5.6265635 | 4.5206146 |
| SCARNA9 | 9.292932 | 6.2073274 | 8.146304 | 7.0421286 |
| RP11-356J5.12 | 8.349426 | 5.703439 | 7.2291236 | 6.1564107 |
| RP5-1171I10.4 | 13.70013 | 10.506689 | 11.589014 | 10.52383 |
| RP11-495P10.1 | 3.7254918 | 7.297061 | 5.807351 | 7.228684 |
| RP11-597D13.9 | 5.928166 | 7.9707985 | 5.124475 | 6.601921 |
| XLOC_009944 | 5.0919933 | 7.8401885 | 4.39557 | 5.9171433 |
| XLOC_001406 | 5.59628 | 7.9149876 | 5.545311 | 7.076109 |
| LINC00520 | 2.4966624 | 5.25226 | 5.7475615 | 7.3201723 |
| RP11-3D4.2 | 4.1100597 | 7.0354123 | 4.659486 | 6.2608733 |
| HOXC-AS5 | 11.2559395 | 13.242913 | 10.212749 | 11.847231 |
| LOC100505815 | 4.59474 | 9.415864 | 7.9784946 | 9.615478 |
| RP11-160C18.2 | 4.189951 | 6.423092 | 5.431453 | 7.0756006 |
| FTCD-AS1 | 4.8723583 | 7.154699 | 2.723096 | 4.379823 |
| RP11-599B13.3 | 7.1518135 | 9.582698 | 6.805849 | 8.493825 |
| CTA-941F9.9 | 6.198222 | 10.469069 | 7.1933956 | 8.890289 |
| RP4-553F4.6 | 3.8593943 | 7.7807436 | 5.177266 | 6.8759227 |
| BC034456 | 2.6104789 | 4.8730865 | 4.6197147 | 6.338605 |
| RNF165 | 4.18044 | 6.3799653 | 5.042949 | 6.7758923 |
| RP11-196G18.3 | 3.972154 | 6.7820296 | 4.783622 | 6.5198345 |
| RP4-564F22.2 | 8.701161 | 10.790463 | 7.6789007 | 9.430214 |
| BC008049 | 5.9439054 | 7.865445 | 5.974086 | 7.7519846 |
| RP4-583P15.10 | 8.032697 | 9.901765 | 8.392622 | 10.212521 |
| BC038194 | 5.110153 | 7.063028 | 3.4031327 | 5.275594 |
| RP11-147I3.1 | 4.836629 | 7.4223742 | 2.990751 | 4.879935 |
| LOC339535 | 2.8128471 | 7.0665016 | 4.9849005 | 6.9553766 |
| LOC728463 | 5.465473 | 7.9039865 | 5.340617 | 7.3371334 |
| AC004383.4 | 9.16566 | 11.446224 | 9.706867 | 11.79911 |
| CTC-210G5.1 | 6.213252 | 8.308514 | 6.2757783 | 8.4018135 |
| RP11-456P18.2 | 5.161702 | 7.634983 | 4.931755 | 7.078368 |
| XLOC_014399 | 5.570559 | 9.589544 | 5.8782125 | 8.0403595 |
| XLOC_011623 | 9.312736 | 11.961374 | 9.803354 | 12.026811 |
| RP11-20I23.8 | 9.479852 | 12.136892 | 9.881562 | 12.12724 |
| FLJ42627 | 9.215469 | 11.758286 | 9.607175 | 11.877242 |
| WASH7P | 7.031228 | 9.438538 | 3.2792957 | 5.6087804 |
| HCG4 | 5.5230484 | 8.668871 | 6.125756 | 8.458603 |
| RP3-523K23.2 | 8.564749 | 11.57539 | 9.548523 | 12.035131 |
| RP4-738P15.1 | 6.6563754 | 8.492294 | 5.2866726 | 7.7935543 |
| AC092296.1 | 5.9729867 | 8.236808 | 4.6712675 | 7.225235 |
| XLOC_000721 | 6.47768 | 8.516439 | 5.4109225 | 7.975415 |
| RP11-706C16.7 | 5.353934 | 7.595755 | 3.9248605 | 6.492882 |
| BC034319 | 6.11842 | 8.596088 | 5.615631 | 8.193882 |
| XLOC_002010 | 4.521965 | 9.073959 | 5.050435 | 7.7430725 |
| AB231723 | 4.619424 | 7.9401546 | 6.417285 | 9.127396 |
| RP11-461A8.4 | 6.5457783 | 8.5014515 | 4.44388 | 7.3332047 |
| RP11-177A2.4 | 6.4182067 | 8.428889 | 4.110711 | 7.006908 |
| CTC-573N18.1 | 6.1647797 | 10.614964 | 7.4744287 | 10.465431 |
| FW340055 | 5.3388696 | 7.9359474 | 6.622889 | 9.717535 |
| RP11-356M20.3 | 4.356319 | 7.08771 | 4.977384 | 8.109167 |
| AX746877 | 9.114954 | 10.957431 | 7.9521456 | 11.202092 |
| RRN3P2 | 6.149128 | 8.156791 | 2.3582609 | 5.72785 |
| RP11-438N16.1 | 5.5740476 | 8.032848 | 5.26862 | 8.870512 |
| CSF2RA | 8.079872 | 9.898769 | 5.8791833 | 10.12546 |
| RP11-320N7.2 | 5.7420926 | 7.948983 | 4.466852 | 9.022362 |
|  |  |  |  |  |
